# Involvement of three putative glucosyltransferases from the UGT72 family in flavonol glucoside/rhamnoside biosynthesis in *Lotus japonicus* seeds

**DOI:** 10.1093/jxb/erw420

**Published:** 2016-11-18

**Authors:** Qinggang Yin, Guoan Shen, Zhenzhan Chang, Yuhong Tang, Hongwen Gao, Yongzhen Pang

**Affiliations:** 1 Key Laboratory of Plant Resources/Beijing Botanical Garden, Institute of Botany, Chinese Academy of Sciences, Beijing 100093, China; 2 University of Chinese Academy of Sciences, Beijing 100049, China; 3 Department of Biophysics, School of Basic Medical Sciences, Peking University, Beijing 100191, China; 4 The Samuel Roberts Noble Foundation, Ardmore, OK 73042, USA; 5 Institute of Animal Science, Chinese Academy of Agricultural Sciences, Beijing 100193, China

**Keywords:** Enzymatic activity, flavonols, *Lotus japonicus*, seed, UDP-glycosyltransferase, UGT72 family.

## Abstract

Flavonols are one of the largest groups of flavonoids that confer benefits for the health of plants and animals. Flavonol glycosides are the predominant flavonoids present in the model legume *Lotus japonicus*. The molecular mechanisms underlying the biosynthesis of flavonol glycosides as yet remain unknown in *L. japonicus.* In the present study, we identified a total of 188 UDP-glycosyltransferases (UGTs) in *L. japonicus* by genome-wide searching. Notably, 12 *UGT*s from the *UGT72* family were distributed widely among *L. japonicus* chromosomes, expressed in all tissues, and showed different docking scores in an *in silico* bioinformatics docking analysis. Further enzymatic assays showed that five recombinant UGTs (UGT72AD1, UGT72AF1, UGT72AH1, UGT72V3, and UGT72Z2) exhibit activity toward flavonol, flavone, and isoflavone aglycones. In particular, UGT72AD1, UGT72AH1, and UGT72Z2 are flavonol-specific UGTs with different kinetic properties. In addition, the overexpression of *UGT72AD1* and *UGT72Z2* led to increased accumulation of flavonol rhamnosides in *L. japonicus* and *Arabidopsis thaliana*. Moreover, the increase of kaempferol 3-*O*-rhamnoside-7-*O*-rhamnoside in transgenic *A. thaliana* inhibited root growth as compared with the wild-type control. These results highlight the significance of the UGT72 family in flavonol glycosylation and the role of flavonol rhamnosides in plant growth.

## Highlight

The UGT72 family is a major group of UDP-glycosyltransferases with diverse substrates. *UGT72AD1*, *UGT72AH1*, and *UGT72Z2* may be involved in flavonol rhamnoside biosynthesis in seeds of *Lotus japonicus*.

## Introduction

Flavonoids, including flavonols, flavones, anthocyanins, isoflavones, and proanthocyanidins, are multifunctional polyphenolic compounds with important implications for plant, animal, and human health (Dixon & Steele, 1999). Flavonoids have a wide variety of bioactivities in human beings, including retarding aging, inhibiting coronary disease, and coordinating hormone levels *in vivo* (Hou, 2003; Kim *et al.*, 2006; Yu *et al.*, 2013). Because of these properties, the bioactivities and potential applications of flavonoids have drawn a great deal of research and commercial attention in recent years.

The attachment of one or more sugar units to different positions of a flavonoid compound can increase its solubility and influence the biological activity of the compound, often increasing its antioxidant and other potential medicinal properties (Chan *et al.*, 2014; Ghanadian *et al.*, 2012; Jones & Vogt, 2001; Kondratyuk & Pezzuto, 2004). Glycosylation is a frequently occurring modification reaction; such reactions are often the last step in the biosynthesis of particular flavonoids. The enzymes that catalyze glycosylation reactions belong to the glycosyltransferase (GT) superfamily, which consists of 98 subfamilies [according to the Carbohydrate-Active enZYmes Database (CAZY); http://www.cazy.org]. Family 1 glycosyltransferases are known to typically transfer a sugar from UDP-glucose donors to small molecules such as hormones, flavonoids, and even pesticides. Consequently, the proteins in this family are also called UDP-glycosyltransferases (UGTs). Although a number of putative *UGT* genes have been identified in the genomes of many plant species, the number of *UGT*s that have been functionally characterized at both the biochemical and molecular genetic levels remains fairly small, and the majority of the information about these proteins comes from studies conducted with *Arabidopsis thaliana* (Caputi *et al.*, 2012).

According to the nomenclature system proposed by the team behind the CAZY website, plant UGT families are numbered from UGT71 to UGT100. All of the *A. thaliana* UGTs are clustered in 21 UGT families (UGT71–76 and UGT78–92; Ross *et al.*, 2001). These UGTs are further phylogenetically clustered into 14 groups (labelled A–N). Among these groups, group E (including the UGT71 and UGT72 families) is the largest, and also appears to have expanded to a greater extent than any of the other groups during the evolution of higher plants (Caputi *et al.*, 2012). However, only a few members of group E have been functionally characterized in *A. thaliana*, including UGT72E1, which is known to function in monolignol glycosylation (Lanot *et al.*, 2006), and UGT72B1, which is known to have both *N*- and *O*-glycosyltranferase activities (Brazier-Hicks *et al.*, 2007). Only a few members of the UGT72 family from other plant species have been shown to have activity toward flavonoids; one such example is the seed coat-specific UGT72L1 from *Medicago truncatula* (Pang *et al.*, 2008). No UGTs have been functionally characterized at the level of a whole family, even the UGT72 family in *A. thaliana.*

Although UGTs often share relatively low sequence identity, their protein tertiary structures are often highly similar, and they all have a GT-B fold. Their N- and C-terminal domains form a cleft that forms the substrate-binding site, and the acceptor mainly binds to the N-terminal domain (Wang, 2009). There is a conserved signature PSPG (plant secondary product glycosyltransferase) motif at the C-terminal end of these proteins. This domain, which consists of 44 amino acids, functions as a nucleoside-diphosphate-sugar binding site (Jadhav *et al.*, 2012). Distinct UGT proteins can utilize various flavonoid aglycones as sugar acceptors. They can glycosylate OH groups at one or more *O*-positions (3, 5, 7, 3ʹ, 4ʹ, or 5ʹ), as well as CH groups at *C*-positions in rice, maize, and buckwheat (Du *et al.*, 2010; Falcone Ferreyra *et al.*, 2013; Nagatomo *et al.*, 2014). As a group, the UGTs have the characteristics of substrate specificity and regioselectivity (He *et al.*, 2006; Jackson *et al.*, 2011; Osmani *et al.*, 2009; Sharma *et al.*, 2014).

*Lotus japonicus*, one of the model legumes used for genetic and genomic studies, is known to accumulate high concentrations of flavonoids. The flavonoid compounds identified in *L. japonicus* are mainly flavonol glycosides; kaempferol glycosides and quercetin glycosides accumulate at relatively high levels in young leaves and seeds (Suzuki *et al.*, 2008). Although several genes involved in the flavonol pathway have been identified in *L. japonicus*, including chalcone isomerase (Shimada *et al.*, 2003), dihydroflavonol 4-reductase (Shimada *et al.*, 2005), and the transcription factors MYB11 and MYB14 (Shelton *et al.*, 2012), few reports have documented the identification and characterization of UGT family proteins in the biosynthesis of flavonol glycosides in this species.

In the present study, using the available genome and transcriptome data resources for *L. japonicus*, we identified a total of 188 putative UGTs, and analyzed the transcription profiles of 71 full-length *UGT* genes. We subsequently focused on the UGT72 family, which has 12 members, and found that three members, UGT72AD1, UGT72AH1, and UGT72Z2, were able to glycosylate flavonols specifically. The genes encoding these three enzymes were found to have seed-specific expression profiles. Taken together, our results provide original information and present an overview of the UGT family in *L. japonicus*. This study sheds light on the functional diversity and biochemical mechanisms of the UGT72 family in flavonoid biosynthesis, and will facilitate comparative functional genomics investigations of the UGTs in legumes and other plants.

## Materials and methods

### Plant materials and chemicals

*L. japonicus* (MG20) plants were grown in an illumination incubator under controlled conditions (16 h/8 h day/night cycle at 25 °C/22 °C, respectively, with a relative humidity of 40%). The roots, stems, leaves, flowers and developing seeds (10, 16, and 20 days after pollination) were collected, immediately frozen in liquid nitrogen, and stored at −80 °C for further use.

Sterile seeds of *A. thaliana* were grown on Murashige and Skoog (MS) medium lacking sucrose, and kept for 5 days in the dark at 4 °C. For the measurement of flavonoid compounds, the seedlings were grown in a tissue culture room maintained at 22 °C with a 16 h/8 h light/dark cycle; light conditions were 100 μmol photons m^−2^·sec^−1^. Seeds were harvested when the plants were completely mature, and were dried at 37 °C for 7 days after harvest. For the measurement of root growth, seedlings were transferred onto plates containing MS medium, and the root lengths were measured for 7 days.

The substrates tested in the present study were purchased from Tongtian Limited Co. (Shanghai, China) and Indofine (Hillsborough, NJ, USA). UDP-glucose and UDP-glucuronic acid were purchased from Sigma-Aldrich (Oakville, CA, USA). Chemicals used in this study were all of analytical or HPLC grade.

### Isolation and analysis of *UGT*s from the UGT72 family in *L. japonicus*

Primers for the 12 *UGT* genes of the UGT72 family were designed according to the published *L. japonicus* genome sequence database. All the forward and reverse primers (see Supplementary Table S6 at *JXB* online) for gene cloning contained different restriction sites. cDNA from leaves of *L. japonicus* was used for gene amplification. The corresponding PCR products were purified and digested using the corresponding restriction enzymes, and then ligated to a pMAL-c2x vector (New England BioLabs, Ipswich, MA, USA) digested with the same restriction enzymes for expression of recombinant protein in *Escherichia coli*.

### Sequence analyses

Predicted protein sequences of *L. japonicus* (data file name: LJpep.bz2, last modified date: 8 November 2010) were retrieved from the PlantGDB (http://www.plantgdb.org; Sato *et al.*, 2008), and further annotated against the protein sequence of *A. thaliana*. The protein sequences of 120 UGTs from *A. thaliana* retrieved from CAZY were used as queries in blastp searches to identify all UGTs from *L. japonicus* (*e*-value<1.00E-5). Multiple sequence alignment of the deduced amino acid sequences was carried out by using DNASTAR. Predicted amino acid sequences of UGTs were aligned using Clustal X2 (Jeanmougin *et al.*, 1998) and used for phylogenetic analysis. The neighbor-joining phylogenetic tree was constructed with 1000 bootstrap replicates using MEGA4.0 software (Tamura *et al.*, 2007).

### Homology modeling and docking statistic

Homology models of the 12 UGTs were built, using the crystal structure of UGT72B1 [Protein Data Bank (PDB) code: 2vg8] as a template, with the SWISS-MODEL server at http://swissmodel.expasy.org (Biasini *et al.*, 2014). The amino acid sequence identities of the 12 UGT72 proteins with UGT72B1 were 45% or greater. UDP-Glc bound in GTB (PDB code: 5c4d) and kaempferol bound in DAPK1 (PDB code: 5aux) were taken as the sugar donor and sugar acceptor for molecular docking. UDP-Glc was docked into the built models of the 12 UGT72 proteins individually, using the Patchdock server at http://bioinfo3d.cs.tau.ac.il/PatchDock/ (Schneidman-Duhovny *et al.* 2005). The model with the highest score for each docked complex was selected for further docking of the sugar acceptor kaempferol, again using the Patchdock program. The model with the highest score for each double-docked complex was selected and visualized with the Pymol molecular graphics system at http://www.pymol.org.

### Expression and purification of recombinant UGT proteins in *E. coli*

The pMAL-UGT expression constructs were transformed into *E. coli* strain *Novablue* competent cells. An aliquot of 0.3 mM isopropyl-β-D-thiogalactoside was added to induce protein expression when the optical density value of the cell culture (grown at 37 °C) reached 0.5. After 24 h incubation at 16 °C with shaking, the cells were harvested by centrifugation at 4 °C and then stored at −80 °C until purification. The MBP-fusion proteins were purified using maltose binding resin according to the pMAL Protein Fusion and Purification System (New England BioLabs).

### Enzyme assay and product identification

The recombinant UGT proteins (5–10 µg) were incubated at 30 °C with 10 mM DTT, 100 mM Tris-HCl (pH 7.5), 0.5 mM substrates, and 4 mM UDP-glucose or UDP-glucuronic acid, in a final volume of 50 µL. Reactions were stopped by the addition of methanol after 30 min, followed by analysis by HPLC after centrifugation at 14000 rpm for 10 min.

For kinetic analysis of the recombinant UGT72AD1, UGT72AH1, and UGT72Z2 proteins, purified enzymes (5–10 µg) were incubated in reaction mixtures comprising 10 mM DTT, 100 mM Tris-HCl (pH 7.5), and 4 mM UDP-glucose, in a final volume of 50 µL. The concentration of the tested acceptor substrates ranged from 0 to 400 µM. Reactions were stopped by the addition of methanol after 30 min incubation at 30 °C. Samples were centrifuged at 14000 rpm for 10 min, and further analyzed by HPLC as previously described (Jiang *et al.*, 2015). The kinetic parameters *K*_m_ and *K*_cat_ were calculated by using the Hyper 32 program (http://hyper32.software.informer.com/).

The flavonol glucoside products were further identified by LC-MS as previously described by Jiang *et al* (2013).

### Expression analysis by quantitative real-time PCR

Total RNA was isolated from roots, stems, leaves, flowers, and developing seeds of *L. japonicus* by using a RNAprep Pure Plant Kit (Tiangen Biotech Co., Beijing, China). RNAs were treated with DNase I (Takara, Japan) to remove any DNA contamination. Reverse transcription was carried out with 1 µg RNA using Moloney Murine Leukemia Virus Reverse Transcriptase (Thermo Fisher Scientific Inc., Waltham, MA, USA). The *UBQ10* gene was used as a housekeeping gene in quantitative real-time reverse transcription PCRs (qRT-PCRs).

Total RNA from hairy roots overexpressing *UGT*s was extracted by the cetyltrimethylammonium bromide method, cleaned with gDNase, and reverse transcribed with the FastQuant RT kit (Tiangen Biotech Co., Beijing, China).

RNAs from transgenic lines of *A. thaliana* overexpressing *UGT*s were extracted by using TRNzol-A^+^ reagent (Tiangen Biotech Co., Beijing, China), followed by reverse transcription after the removal of genomic DNA contamination. The *P2PA* gene was used as a housekeeping gene used for these qRT-PCRs.

In all cases the PCR conditions were as described previously by Jiang *et al* (2015). Primer sequences used for qRT-PCR are listed in Supplementary Table S6. Data were calculated from three biological replicates, and each biological replicate was examined in triplicate.

### Treatment of *L. japonicus* hairy roots with salicylic acid, abscisic acid, and jasmonic acid

Hairy roots of *L. japonicus* induced by ARqual1 with pCXSN empty vector were maintained in 50 mL B5 liquid medium with 250 mg·L^−1^ timentin and carbenicillin for 4 weeks. The hairy roots were collected and divided into six aliquots; each aliquot was transferred into a new 100 mL Erlenmeyer flask with fresh medium. After overnight culture at 25 °C with rotation (100 rpm) under diffuse light, one aliquot was used as an untreated control; the other five aliquots were treated with salicylic acid (SA; 100 μM), abscisic acid (ABA; 50 μM) and Me-jasmonic acid (JA; 100 μM) for 2, 4, 8, 12, and 24 h, respectively. All hairy roots were harvested and immediately frozen in liquid nitrogen, and stored at −80 °C until use.

### Overexpression of *UGT72AD1* and *UGT72Z2* in *L. japonicus* hairy roots and *A. thaliana*

The ORF region of the *UGT72AD1* and *UGT72Z2* genes driven by the 35S CaMV promoter was cloned to the binary vector pCXSN for gene overexpression *in planta*. The resulting pCXSN-UGT72AD1 and pCXSN-UGT72Z2 constructs were transformed into *Agrobacterium* strains GV3101 and ARqual1, and used to generate transgenic *A. thaliana* (GV3101) and hairy roots of *L. japonicus* (ARqual1). The hairy roots of *L. japonicus* were maintained on B5 agar medium supplemented with 10 mg·L^−1^ hygromycin and antibiotics (250 mg·L^−1^ timentin and carbenicillin).

### Analysis of flavonol metabolites

The *L. japonicus* hairy root lines with higher gene expression levels were selected and extracted for flavonol analysis on HPLC. Seeds of T3 generation homozygous transgenic *A. thaliana* overexpressing *UGT72AD1* and *UGT72Z2* were screened by growth on MS medium supplemented with hygromycin (30 mg·L^−1^) under dark conditions for 5 days at 23 °C. The 3-week-old leaves and mature seeds from both transgenic and wild-type control *A. thaliana* plants were collected for flavonol analysis on HPLC.

The extraction of flavonol metabolites was performed as described previously (Yin *et al.*, 2012) with minor modifications. Briefly, 5 μg dry weight of ground *L. japonicus* hairy roots and *A. thaliana* seedlings was extracted with 0.5 mL methanol (10 μM naringenin was used as an internal standard) for 1 h with rotation (140 rpm) at 4 °C. The extracts were subsequently separated by centrifugation at 14000 rpm for 10 min at 4 °C. One-third volume of distilled water was added to the supernatant, and 20 µL of the cleared extract was analyzed on HPLC after another centrifugation at 14000 rpm for 20 min.

## Results

### Screening of *UGT* genes in the *L. japonicus* genome

To identify putative *UGT* genes in the *L. japonicus* genome, an implementation of the BLAST algorithm was used to search the whole genome sequence; 120 *UGT*s from *A. thaliana* were used as queries in this analysis. Using this method, 188 putative *UGT* genes were identified in the *L. japonicus* genome. *L. japonicus* thus appears to have 68 more putative *UGT*s than does *A. thaliana*; the true number of *UGT*s in *L. japonicus* may differ from this number, owing to incomplete coverage of the genome sequence (Sato *et al.*, 2008). A summary of the identification numbers, orthologs in *A. thaliana*, identity percentages, and BLAST *e*-values for the 188 putative *L. japonicus UGT* genes is presented in Supplementary Table S1.

Sequence analysis revealed that 71 of the putative *L. japonicus UGT*s were full-length genes, 70 were partial genes, and 47 were considered to be pseudogenes. The physical locations of 63 of the 71 full-length genes were assigned to the six chromosomes of the *L. japonicus* genome. The remaining eight were assigned to an as yet unattributed scaffold (Supplementary Fig. S1). The distribution of the putative *UGT* genes on the chromosomes appeared to be even, although relatively higher densities of *UGT* genes were found in some locations on chromosomes 1 and 3 compared with other locations (Supplementary Fig. S1).

**Fig. 1.**
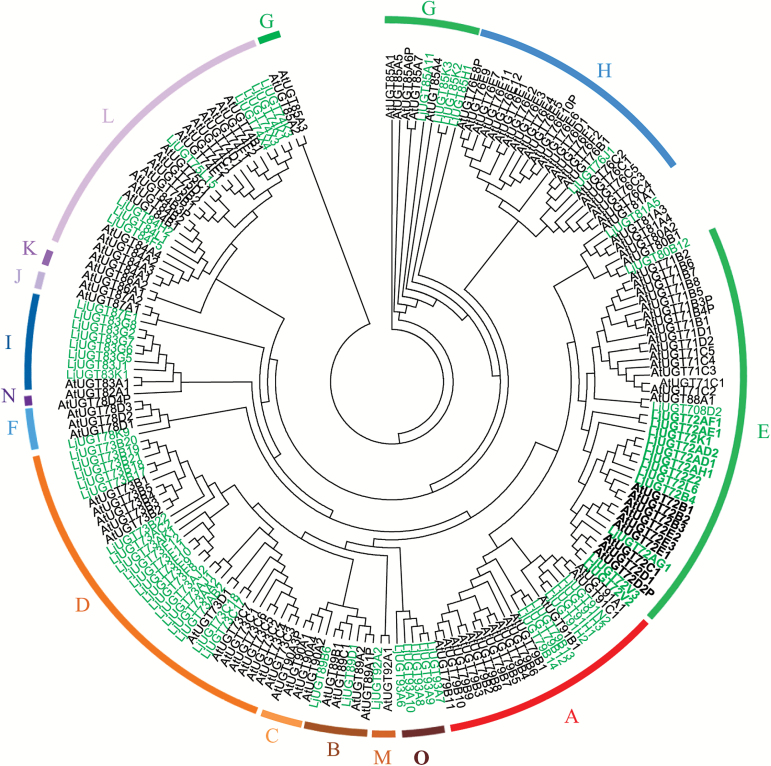
**Phylogenetic analysis of UGTs from *L. japonicus* and *A. thaliana*.** Amino acid sequences were analyzed using the Clustal X2 program and the bootstrap consensus tree was constructed using MEGA 4.0 software with the neighbor-joining method and 1000 bootstrap replicates. The respective protein names and numbers are listed in Supplementary Table S1. UGTs from *L. japonicus* are indicated in green and those from *A. thaliana* are in black. The 15 groups are indicated in different colors. (This figure is available in colour at *JXB* online.)

Furthermore, we obtained, with the help of the UGT nomenclature committee, the nomenclature-appropriate names of 71 full-length *UGT* genes that had signature PSPG motifs (Supplementary Table S2). We constructed a phylogenetic tree with these 71 full-length *UGT* genes and with all 120 *UGT* genes from *A. thaliana* (Fig. 1). Analysis of the phylogenetic tree revealed that the 71 *UGT*s from *L. japonicus* were clustered into 19 families. Alternatively, using the scheme previously employed for *A. thaliana* (Ross *et al.*, 2001), the *L. japonicus UGT*s can be clustered into 14 groups (A–N), albeit with one additional group (O) (Fig. 1). The UGT73 family, with 19 members in group D, and the UGT72 family, with 12 members in group E, are the two largest families in *L. japonicus* (Fig. 1). Members of the UGT73 family have been studied extensively in many other plant species. For this reason, we focused mainly on the less well-documented UGT72 family for functional characterization experiments in the present study.

### Expression profiles of *UGT* genes in *L. japonicus*

**Fig. 2.**
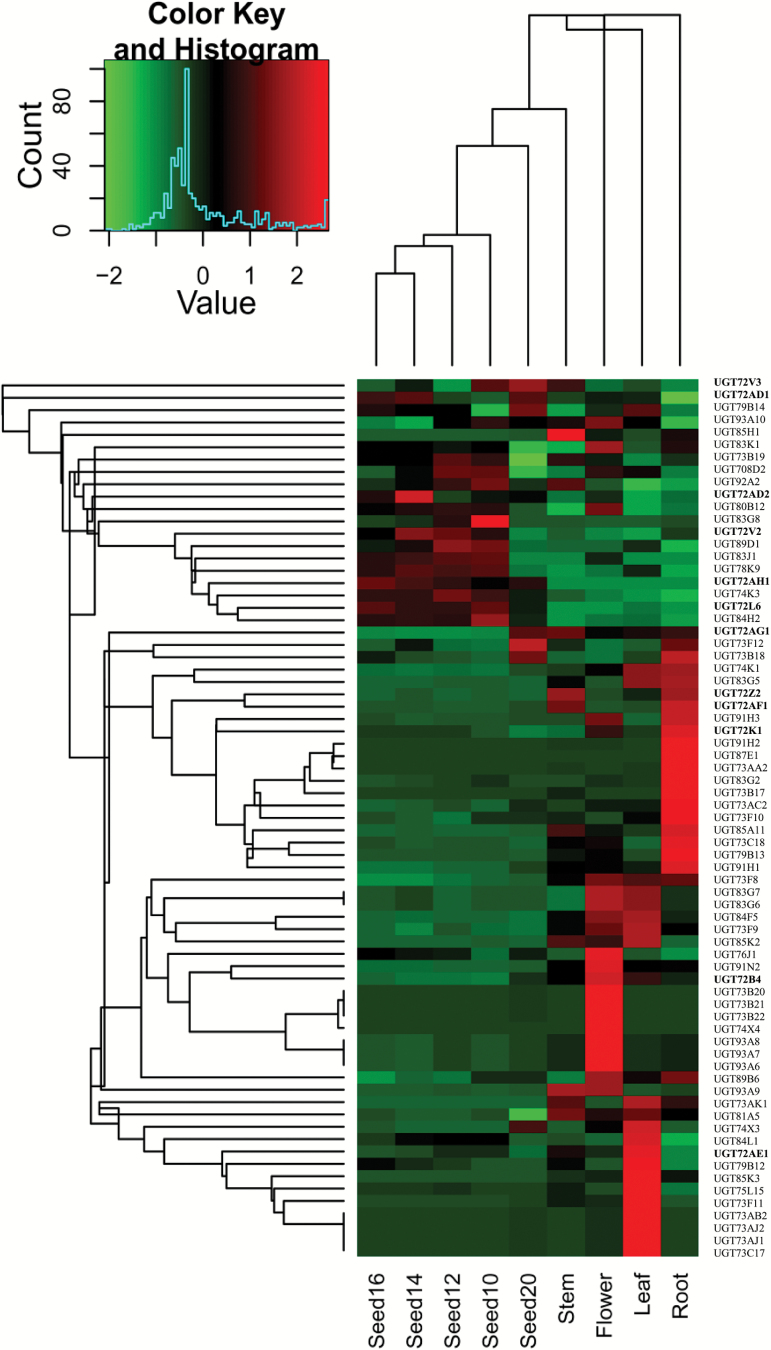
**Hierarchical clustering analysis of transcript levels of *UGT*s in different tissues and seed development stages of *L. japonicus*.** Data were retrieved from the *L. japonicus* gene expression atlas, and the clustering was performed using Heatmap2. Sample details are described by Verdier *et al* (2013). The heat map shows the relative expression level of each gene in various tissues. The color scale (–2 to 2 in green to red) represents *Z*-score normalized gene expression from the genechip data. Dendrograms along the top and left sides of the heat map indicate the hierarchical clustering of tissues and genes. (This figure is available in colour at *JXB* online.)

The expression profiles of 71 full-length *UGT* genes were retrieved from the *L. japonicus* gene expression atlas (Verdier *et al.*, 2013; Supplementary Table S2). A hierarchical clustering analysis of the transcript data indicated that 15, 12, 10, and 10 *UGT* genes were specifically expressed in only one tissue (in developing seeds, roots, flowers, or leaves, respectively) (Fig. 2).

Among the *UGT72* family genes, *UGT72AD1*, *UGT72AH1*, *UGT72V3*, *UGT72AD2*, *UGT72V2*, and *UGT72L6* were preferentially expressed in seeds (Fig. 2). *UGT72AG1*, *UGT72AE1*, *UGT72B4*, and *UGT72K1* were preferentially expressed in stems, flowers, and roots, respectively (Fig. 2). *UGT72AF1* and *UGT72Z2* were expressed strongly in both stems and roots. Overall, the *L. japonicus* UGT72 family members exhibited differential and diverse expression profiles, implying that they have differentiated biological functions in various tissues.

### Sequence and docking analyses of 12 members of the UGT72 family in *L. japonicus*

In order to further characterize the 12 members of the UGT72 family, their nucleotide sequences were amplified from cDNA prepared from *L. japonicus* leaves. Their open reading frames ranged from 1410 to 1551 bp in length, encoding deduced proteins ranging from 470 to 516 amino acids. Most of these *UGT72* genes did not have introns, but *UGT72AD2* and *UGT72AH1* had introns of 355 bp and 2139 bp, respectively. Amino acid sequence analysis showed that these proteins shared 32–84% identity (Supplementary Table S3). UGT72A1 and UGT72A2 (79% identity), and UGT72V2 and UGT72V3 (84% identity), shared relatively high sequence identity in comparison to other relationships among the 12 protein sequences. Rather than sharing high intra-species sequence identity, most of the UGT72 family members had their highest sequence identity with an ortholog from another legume species, such as *Glycine soja, Glycine max*, *Pueraria montana* var. lobata, *Cicer arietinum*, *M. truncatula*, or *Phaseolus vulgaris* (61–78% identity; Supplementary Table S4).

**Fig. 3.**
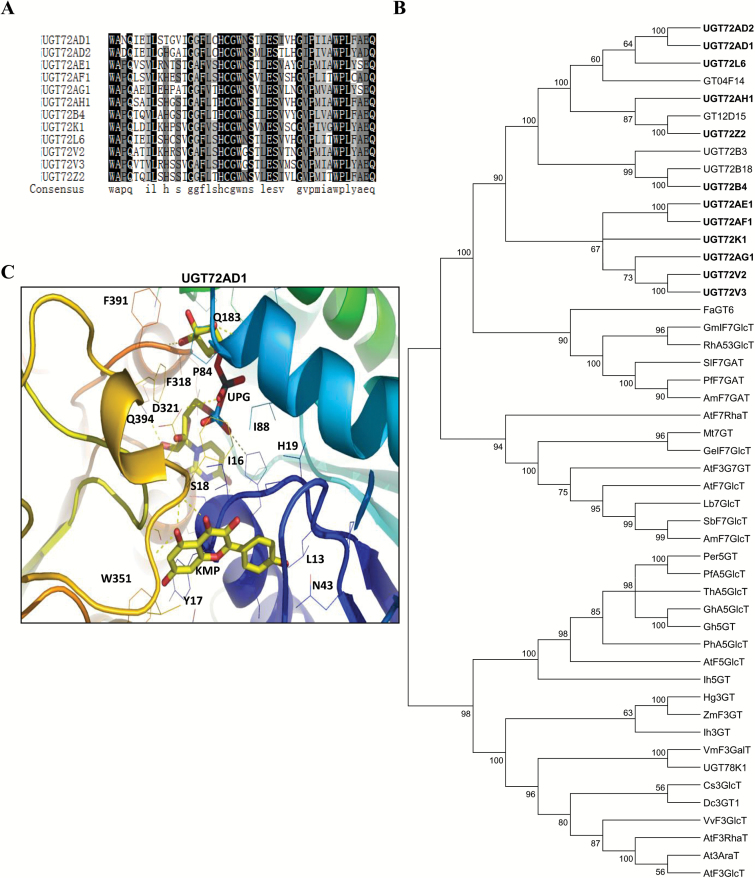
**Sequence alignment of PSPG motifs and phylogenetic analysis of UGTs.** (A) Multiple sequence alignment of PSPG motifs of 12 UGTs. Identical residues are highlighted with a black background and similar residues with a gray background. (B) Phylogenetic relationship of flavonoid UGTs. The tree was constructed as described in the Materials and methods. Protein sequences were aligned with Clustal X2 and a neighbor-joining tree was constructed using MEGA4.0 software. (C) Structural model of one representative UGT (UGT72AD1) docked with UDP-glucose and a flavonol (kaempferol). (This figure is available in colour at *JXB* online.)

Multiple sequence alignment revealed that the 12 *UGT*s shared a conserved domain with the PSPG motif (Fig. 3A) near their C-terminal domain (Supplementary Fig. S2). The last glutamine (Q) residue within the PSPG motif is thought to confer specificity for UDP-glucose as the sugar donor (Kubo *et al.*, 2004). Notably, all 12 UGTs possess this Q, suggesting they may all use UDP-glucose as a sugar donor.

An unrooted phylogenetic tree showed that these 12 members of the UGT72 family were clustered into a single clade containing two UGTs (GT04F14 and GT12D15) from *P. montana* var. lobata, one UGT (UGT72B3) from *A. thaliana*, and one (UGT73B18) from *C. arietinum* (Fig. 3B). The UGT72A1 and UGT72A2 pair and the UGT72V2 and UGT72V3 pair were closely clustered together. In addition, they were close to a cluster comprising FaGT6, GmIF7GT, RhA53GlcT, SlF7GalT, PfF7GalT, and Am7RhaT; these UGTs have been demonstrated to have activity with various flavonoid substrates (Fig. 3B). The UGT72 family of 12 UGTs from *L. japonicus* was separated from three other clusters with UGTs that displayed glycosylation activities at 7-OH, 5-OH, or 3ʹ-OH positions, implying that the UGT72 family is different from other known families of UGTs.

To determine the molecular basis for the specificity of the 12 *L. japonicus* UGT72 enzymes, three-dimensional structure models in which the enzymes were docked with UDP-glucose and a flavonol substrate (kaempferol) were constructed using the Patchdock program. The docking statistics of UGT72AH1, UGT72L6, UGT72Z2, and UGT72AD1 had relatively higher scores among the 12 proteins (more than 4000; Supplementary Table S5), suggesting that these four proteins are more likely to be active toward kaempferol. In contrast, UGT72AF1, UGT72V2, UGT72B4, and UGT72AG1 showed lower scores among the 12 proteins, suggesting that they are unlikely to be active toward kaempferol (less than 3900; Supplementary Table S5).

All 12 UGTs were subjected to analysis of the potential interactions between amino acid residues and the substrates/UDP-glucose (Supplementary Fig. S3A). Details for four of the 12 UGTs (UGT72L6, UGT72AD1, UGT72V3, and UGT72AF1) are shown in Fig. 3C and Supplementary Fig. S3B. Six amino acids for each of UGT72AD1 (S18, H19, P84, Q183, D321, and Q394) and UGT72V3 (L19, H21, S275, Y314, Y387, and E389) were predicted to interact with the sugar donor UDP-glucose (Fig. 3C and Supplementary Fig. S3B). In contrast, only three amino acids for each of UGT72AF1 (N153, K175, and K181) and UGT72L6 (S248, D252, and R255) were predicted to interact with UDP-glucose (Fig. 3C and Supplementary Fig. S3B). The ligand-binding sites for UGT72AD1 and UGT72V3 were predicted to be in the central cleft formed by the N- and C- terminal domains (Supplementary Fig. S3C), whereas those for UGT72AF1 and UGT72L6 were predicted to be far away from the central region (Supplementary Fig. S3C). These findings suggest that UGT72AD1 and UGT72V3 are more likely to accept kaempferol as a substrate than UGT72AF1 or UGT72L6, consistent with their *in vitro* enzymatic activities as described below.

### Enzymatic activity of UGT72 family proteins in *L. japonicus*

To further investigate the *in vitro* activity of UGT72 family enzymes, 12 UGTs were expressed in *E. coli*, and the recombinant proteins were evaluated with enzymatic assays (Supplementary Fig. S4). Two sugar donors (UDP-glucose and UDP-glucuronic acid) and 13 flavonoid aglycones (Supplementary Table S5) were tested as substrates.

Five of the recombinant UGTs (UGT72AD1, UGT72AF1, UGT72AH1, UGT72V3, and UGT72Z2; Supplementary Fig. S4) exhibited catalytic activity toward flavonoid aglycones with UDP-glucose but not UDP-glucuronic acid as sugar donor. UGT72AD1, UGT72AH1, and UGT72Z2 exhibited specific activity toward only flavonol aglycones (kaempferol, quercetin, and myricetin), consistent with their high docking scores for these compounds (Supplementary Table S5). UGT72AF1 exhibited activity toward apigenin, daidzein, and genistein, while UGT72V3 showed activity toward kaempferol, quercetin, myricetin, lutiolin, genistein, and daidzein (Supplementary Table S5). No activity was detected for the remaining seven recombinant UGT proteins with any of the tested substrates, regardless of whether the sugar donor for the assay was UDP-glucose or UDP-glucuronic acid (Supplementary Table S5).

**Fig. 4.**
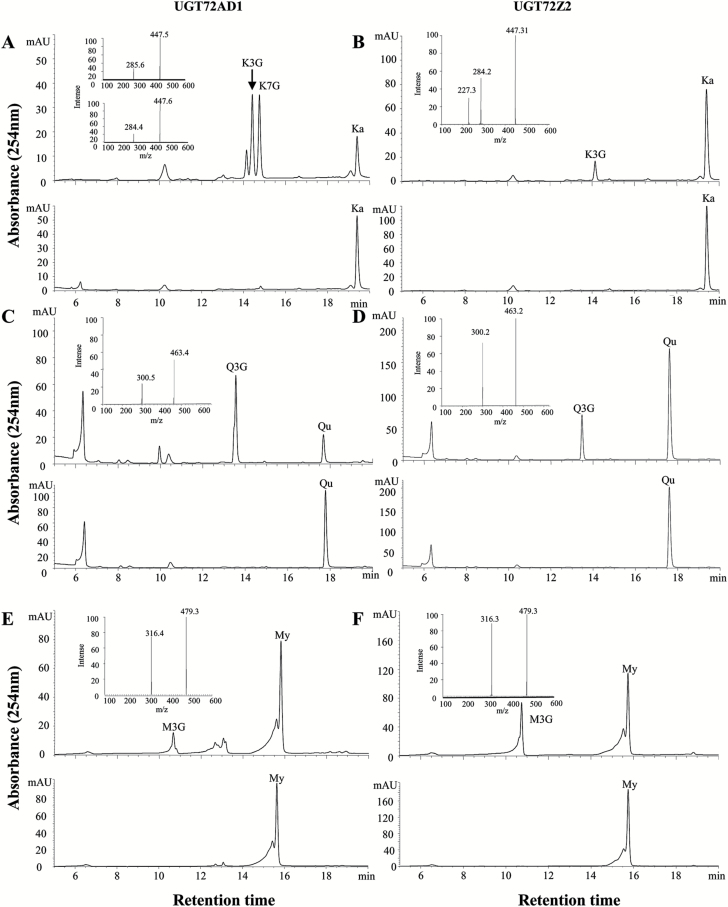
**Analysis of UGT72AD1 and UGT72Z2 enzymatic reaction products.** (A, B) Representative HPLC chromatograms of (A) UGT72AD1 and (B) UGT72Z2 with kaempferol (Ka) as substrate (upper panels). The reactions with denatured proteins were used as negative controls (lower panels). In (A), insertions indicate the mass spectrum of kaempferol 3-*O*-glucoside (upper panel) and kaempferol 7-*O*-glucoside (lower panels) for UGT72AD1. In (B), the insertion indicates the mass spectrum of kaempferol 3-*O*-glucoside for UGT72Z2. (C, D) Representative HPLC chromatograms of (C) UGT72AD1 and (D) UGT72Z2 with quercetin (Qu) as substrate (upper panels). The reactions with denatured proteins were used as negative controls (lower panels). Insertions indicate the mass spectrum of quercetin 3-*O*-glucoside for both UGT72AD1 and UGT72Z2. (E, F) Representative HPLC chromatograms of (E) UGT72AD1 and (F) UGT72Z2 with myricetin (My) as substrate (upper panels). The reactions with denatured proteins were used as negative controls (lower panels). Insertions indicate the mass spectrum of myricetin 3-*O*-glucoside for both UGT72AD1 and UGT72Z2. In all the enzymatic reactions, UDP-glucose was used as the donor substrate. The other peaks without name labels indicate UV absorption by products.

Analysis of the enzymatic products by HPLC showed that assays with recombinant UGT72AD1 and UGT72Z2 (as for UGT72AH1) produced new products with kaempferol (Fig. 4A, B), quercetin (Fig. 4C, D), and myricetin (Fig. 4E, F), compared with their corresponding controls (Fig. 4A–F, lower panels). These products were analyzed via HPLC coupled with UV and electrospray ionization mass spectrometry, which revealed that all of the compounds were characterized by the loss of one glucose (162) moiety to yield the corresponding aglycones with the three substrates (Fig. 4A–F, insertions), indicating that they were all flavonol monoglucosides. Comparison with authentic reference standards further verified that these compounds were all flavonol 3-*O*-monoglucosides (K3G, Q3G, and M3G; Fig. 4A–F, upper panels). The assay with recombinant UGT72AD1 produced two monoglucosides with kaempferol as the substrate (Fig. 4A); they were identified as kaempferol 3-*O*-glucoside and kaempferol 7-*O*-glucoside based on comparison of their MS spectrum with those of authentic reference standards.

**Fig. 5.**
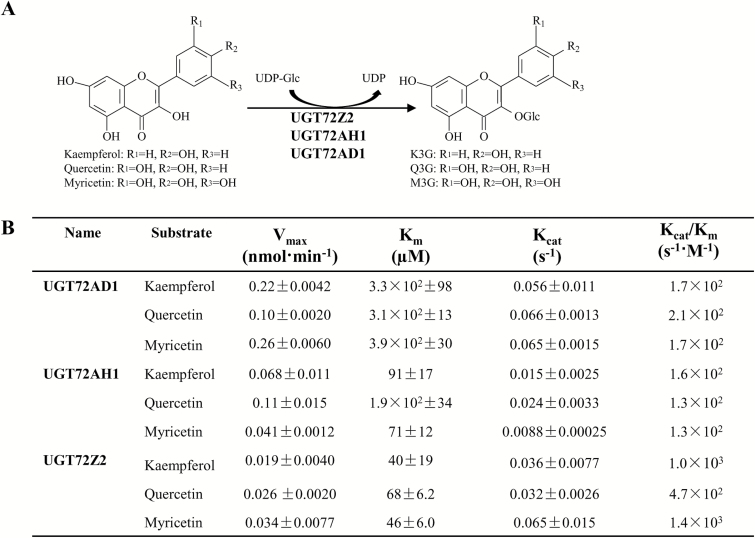
**Enzyme kinetics of recombinant UGT72AD1, UGT72AH1 and UGT72Z2 proteins.** (A) Chemical structures of flavonol substrates and products catalyzed by the three recombinant proteins. (B) Kinetic parameters of the recombinant UGT72AD1, UGT72AH1, and UGT72Z2 proteins with flavonol aglycones as acceptor substrates and UDP-glucose as donor substrate. Values represent the means±SD from triplicate enzymatic assays.

Recombinant UGT72AD1, UGT72AH1, and UGT72Z2 proteins exhibited different *K*_m_, *V*_max_, and *K*_cat_ parameters (Fig. 5). Notably, UGT72Z2 exhibited substantially higher affinity (lower *K*_m_) for flavonol aglycones (*K*_m_ values of 40 µM, 68 µM, and 46 µM for kaempferol, quercetin, and myricetin, respectively) than either UGT72AH1 or UGT72AD1 (with *K*_m_ values of 91 µM, 190 µM, and 71 µM for UGT72AH1, and 330 µM, 310 µM, and 390 µM for UGT72AD1 toward kaempferol, quercetin, and myricetin, respectively) (Fig. 5A, B).

### Expression patterns of *UGT72AD1*, *UGT72AH1*, and *UGT72Z2*

**Fig. 6.**
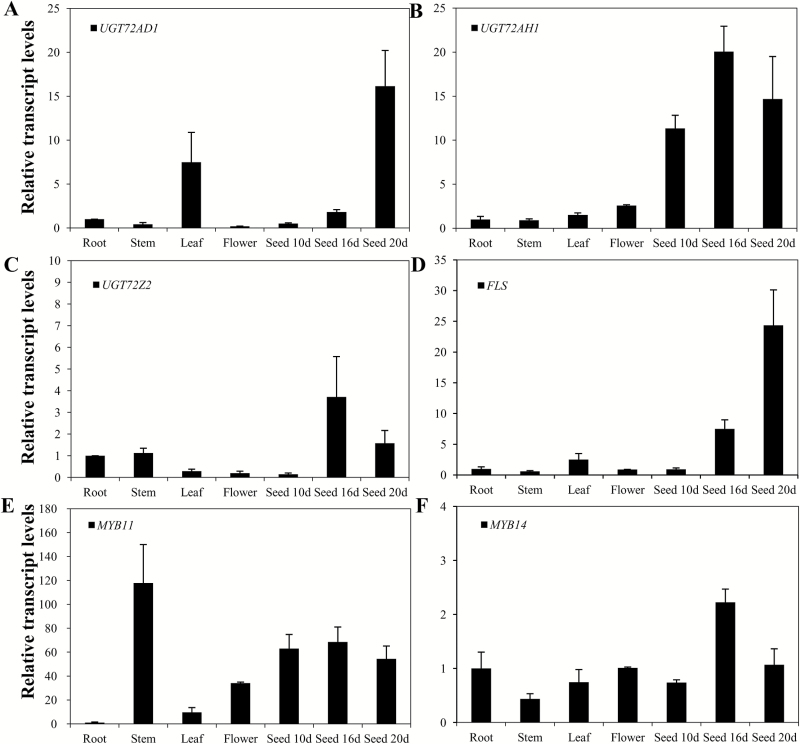
**Expression profiles of *UGT72AD1*, *UGT72AH1*, and *UGT72Z2* with *FLS*, *MYB11*, and *MYB14* in various tissues of *L. japonicus*.** Relative transcript levels of (A) *UGT72AD1*, (B) *UGT72AH1*, (C) *UGT72Z2*, (D) *FLS*, (E) *MYB11*, and (F) *MYB14* in roots, stems, leaves, flowers, 10-day seeds, 16-day seeds, and 20-day seeds, as assessed by qRT-PCR analysis (d, days after pollination). The transcript level of each gene in root tissue was set at a value of 1. Values represent the means±SD from triplicate samples.

In order to further characterize the functions of UGT72AD1, UGT72AH1, and UGT72Z2, which possess specific enzymatic activity toward flavonols, their expression levels in various tissues were analyzed by qRT-PCR. For *UGT72AD1*, the highest transcript level was found in seeds at the later stage of development (20 days after pollination) among all the tested tissues (Fig. 6A). *UGT72AH1* was more highly expressed in seeds than in roots, stems, leaves, or flower tissues (Fig. 6B). *UGT72Z2* had its highest transcript level in seeds 16 days after pollination (Fig. 6C). The highest transcript levels of these three *UGT* genes were all detected in the later stages of seed development (16 or 20 days after pollination), implying that these are seed-specific *UGT* genes which function primarily in seeds.

The expression profile of *FLS* and two major transcription factors, *MYB11* and *MYB14*—three genes that are known to be involved in flavonoid biosynthesis—were analyzed in parallel with the abovementioned three *UGT* genes. *FLS* was highly expressed in 20-day and 16-day seeds (Fig. 6D). For *MYB11*, the transcript levels in seeds were higher than those in leaves or flowers (Fig. 6E). The transcript levels of *MYB14* were slightly higher in 16-day seeds than in the other tissues analyzed (Fig. 6F). Considered together, the similar transcript profiles of *UGT72AD1* and *FLS* suggested that these two genes may be regulated in a coordinated fashion by *MYB11* for flavonol biosynthesis in *L. japonicus* seeds.

### Effects of SA, ABA, and JA on expression of *UGT72AD1*, *UGT72AH1*, and *UGT72Z2*

In a previous study, the expression of two flavonol *O*-glucosyltransferases from strawberry, *FaGT6* and *FaGT7*, was found to be induced by SA treatment (Griesser *et al.*, 2008). In the present study, in order to investigate whether the expression of the three flavonol-specific *UGT*s was affected by SA, ABA, and/or JA, hairy roots of *L. japonicus* were used for a series of treatments. RT-PCR and qRT-PCR methods were used to measure the expression levels of *UGT72AD1*, *UGT72AH1*, and *UGT72Z2*, as well as two transcription factors, *MYB11* and *MYB14*. *UGT72AD1* expression was almost undetectable in *L. japonicus* hairy roots, even in samples treated with SA, JA, and ABA, suggesting that *UGT72AD1* is unlikely expressed in hairy roots and that its expression is not induced by SA, JA, or ABA treatment.

Treatment of *L. japonicus* hairy roots with SA induced transcription of *UGT72AH1* by more than 28-fold at 4 h and 12-fold at 8 h compared with untreated controls. Transcription of *UGT72Z2* was induced with SA by more than 28-fold at 12 h (Supplementary Fig. S5A). The expression of *MYB11* and *MYB14* showed the same trend as *UGT72Z2* (Supplementary Fig. S5A). The total flavonoid content increased from 0 h to 2 h, and then remained at the same level until the 24 h time point (Supplementary Fig. S5B). Flavonol hexoside (F3-2-2) and total flavonol content followed a similar trend, with increases observed at the 2 h and 4 h time points (Supplementary Fig. S5C).

ABA treatment did not lead to significantly increased expression of *UGT72AH1*, *UGT72Z2*, or *MYB14* (Supplementary Fig. S5A). *MYB11* expression did increase in treated plants, with the highest level observed at 2 h (14-fold increase over untreated controls); its expression in treated roots was 10-fold higher than that in untreated controls at 24 h (Supplementary Fig. S5A). Similarly, neither the total flavonol content nor the accumulation of three individual flavonol hexosides (F3-2-2, F2, and F2-1) changed significantly with ABA treatment (Supplementary Fig. S5B, C).

JA treatment induced the expression of *UGT72Z2* quickly, by more than 10-fold compared with untreated controls at 2 h (Supplementary Fig. S5A). *MYB11* expression increased slightly throughout the JA treatment period (Supplementary Fig. S5A). Accordingly, the total flavonoid content followed a similar trend to that of the transcript level of *UGT72Z2*, with the highest increase observed at 24 h (81% higher than untreated controls; Supplementary Fig. S5B). JA treatment did not significantly alter the accumulation of the three major flavonol glycosides (Supplementary Fig. S5C).

Taken together, these results indicated that the expression levels of *UGT72AH1*, *UGT72Z2*, *MYB11*, and *MYB14* responded differently to the SA, ABA, and JA treatments. In particular, the obvious induction of *UGT72AH1* and *UGT72Z2* by SA treatment suggested that these two genes may play a role in plant defense responses.

### Overexpression of *UGT72AD1* and *UGT72Z2* in *L. japonicus* hairy roots

UGT72AD1, with the highest *K*_m_ for flavonol substrates, and UGT72Z2, with the lowest *K*_m_, were used as representatives of the UGT72 family to further investigate their *in vivo* function in transgenic *L. japonicus* hairy roots. The expression of *UGT72AD1* and *UGT72Z2* in transgenic hairy roots was analyzed with qRT-PCR (Supplementary Fig. S6A). Three out of more than 20 transgenic lines that had higher transcript levels for each gene were used for further analyses.

The total flavonoid content did not change significantly in any transgenic lines in comparison to the wild-type control (Supplementary Fig. S6B). The flavonol glycosides that accumulated in hairy roots with the two transgenes included kaempferol 3-*O*-rhamnoside-7-*O*-rhamnoside (F1) and kaempferol 3-*O*-glucoside-7-*O*-rhamnoside (F2), and an additional two deduced flavonol hexosides (F3-2-2 and F2-1) (Supplementary Figs S6C, S7).

The amount of kaempferol 3-*O*-rhamnoside-7-*O*-rhamnoside increased slightly in *UGT72AD1*-overexpressing lines 5 and 6 (69% and 10%; Supplementary Fig. S6C) compared with the wild-type control. The levels of kaempferol 3-*O*-glucoside-7-*O*-rhamnoside increased from 19% to 187% in *UGT72AD1*-overexpressing lines compared with the wild-type control. In *UGT72Z2*-overexpressing lines, the levels of kaempferol 3-*O*-glucoside-7-*O*-rhamnoside increased from 49% to 108% compared with the wild-type control (Supplementary Fig. S6C, *P*<0.05). In addition, the amounts of the two deduced flavonol hexosides (F3-2-2 and F2-1) and the total flavonol glucoside content increased in all six overexpression lines compared with the wild-type control, with the highest content found in line 5 for *UGT72AD1* and line 6 for *UGT72Z2* (Supplementary Fig. S6C). Taken together, the increase of flavonol glucosides indicated that both UGT72Z2 and UGT72AD1 could transfer sugars (glucose and rhamnose) to flavonol substrates in *L. japonicus* hairy roots.

### Ectopic expression of *UGT72AD1* and *UGT72Z2* in *A. thaliana*

**Fig. 7.**
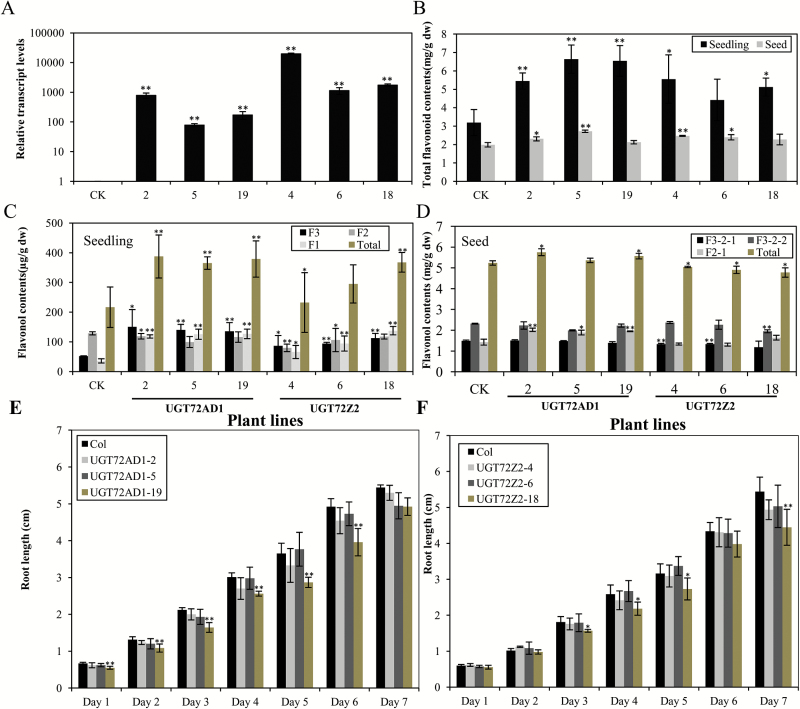
**Overexpression of *UGT72AD1* and *UGT72Z2* genes in *A. thaliana*.** (A) Relative transcript levels of *UGT72AD1* and *UGT72Z2* genes in transgenic and wild-type (CK) *A. thaliana* detected by qRT-PCR analysis. (B) Total flavonoid contents in seedlings and seeds of transgenic *A. thaliana* lines overexpressing *UGT72AD1* and *UGT72Z2* genes and in a wild-type control (CK). (C, D) Flavonol contents in (C) seedlings and (D) seeds of transgenic *A. thaliana* lines overexpressing *UGT72AD1* and *UGT72Z2* genes and in a wild-type control (CK). (E, F) Root length of *A. thaliana* seedlings overexpressing (E) *UGT72AD1* and (F) *UGT72Z2* genes, and of a wild-type control (CK). Values represent the means±SD of triplicate analytical replicates from independent transgenic lines and control. Data were statistically evaluated using Student’s *t* test (**P<0.01, * *P*<0.05). (This figure is available in colour at *JXB* online.)

To evaluate whether UGT72AD1 and UGT72Z2 were functional in other plant species, the genes encoding these two proteins were ectopically expressed in *A. thaliana*, a species that is abundant in flavonols. The transcript levels in transgenic *A. thaliana* lines overexpressing *UGT72AD1* or *UGT72Z2* were confirmed by qRT-PCR. Three lines with high expression levels for each gene were used for flavonoid analysis (Fig. 7A).

The total flavonoid content increased in seedlings overexpressing either *UGT72AD1* or *UGT72Z2* (Fig. 7B). In particular, the total flavonoid content almost doubled in line 5 overexpressing *UGT72AD1* (Fig. 7B). However, the total flavonoid content in the seeds of these transgenic *A. thaliana* plants was not significantly different from that of wild-type *A. thaliana* (Fig. 7B).

A previous study reported that seedlings of wild-type *A. thaliana* (ecotype Columbia-0) accumulate three major flavonol glycosides, including kaempferol 3-*O*-rhamnoside-7-*O*-rhamnoside (F1), kaempferol 3-*O*-glucoside-7-*O*-rhamnoside (F2), and kaempferol-3-*O*-[rhamnosyl(1→2glucoside)]-7-*O*-rhamnoside (F3) (Yin *et al.*, 2012; Supplementary Fig. S8). In comparison with wild-type *A. thaliana*, the levels of F1 increased between 82% and 294% in five transgenic lines (lines 2, 5, 19, 6, and 18) (Fig. 7C). In contrast, the levels of F2 did not increase, relative to the control, in any of the six lines (Fig. 7C). The levels of F3 increased by 211%, 190%, and 185% in *UGT72AD1*-overexpressing lines 2, 5, and 19, and by 64%, 91%, and 127% in *UGT72Z2*-overexpressing lines 4, 6, and 18, respectively, relative to the wild-type control (Fig. 7C). The increase in levels of F3, which has a glucose moiety, indicated that UGT72AD1 and UGT72Z2 could transfer glucose to kaempferol, and that these enzymes were functional *in vivo* in *A. thaliana*. Additionally, the increased accumulation of these glycosides with rhamnose moieties suggested that both UGT72AD1 and UGT72Z2 most likely utilize UDP-rhamnose as a sugar donor in *A. thaliana*. However, the total flavonol content, and the content of the three flavonol glycosides (F1, F2 and F3), were not significantly different in seeds of transgenic *A. thaliana* compared with the wild-type control (Fig. 7D, Supplementary Fig. S9).

In order to evaluate the effects of the changes in flavonol glycoside accumulation on plant growth, transgenic *A. thaliana* seedlings overexpressing *UGT72AD1* and *UGT72Z2* were used for root length growth assays. Significant inhibition of root length was observed in one *UGT72AD1*-overexpressing line (line 19) and one *UGT72Z2*-overexpressing line (line 18; Fig. 7E, F), consistent with the significant increase in levels of kaempferol 3-*O*-rhamnoside-7-*O*-rhamnoside in these two lines. This result indicated that overexpression of *UGT72AD1* and *UGT72Z2* affected root growth in *A. thaliana*.

## Discussion

Flavonols are one of the largest groups of flavonoids. They can specifically protect the photosynthetic tissues of plants from damage by UV radiation (Martinez-Luscher *et al.*, 2014; Rodov *et al.*, 2010). Flavonols are also potent antioxidants and major components of many medicinal plants (Biscaro *et al.*, 2013; Dayem *et al.*, 2015; Manach *et al.*, 2005; Scalbert *et al.*, 2005; Williamson & Manach, 2005). Glycosylated flavonols, as the major form of flavonols in plants, contribute greatly to plant growth and development, as well as to human health (Babu *et al.*, 2013; Ringli *et al.*, 2008).

Due to the heterogeneity of UGTs for glycosylation in plants, the function of UGTs for the biosynthesis of specific flavonol glycosides remains largely unknown in many plant species. For example, although flavonol glycosides have been found to be remarkably abundant in *L. japonicus* (Suzuki *et al.*, 2008), no *UGT* gene has as yet been comprehensively characterized in this model legume species.

### Identification and characterization of *UGT* genes in *L. japonicus*

Our genome-wide search of *UGT* genes identified 188 putative genes for UGTs in *L. japonicus*, a number that is comparable to the 187, 125, and 242 *UGT* genes in the other model legumes *M. truncatula*, *C. arietinum*, and *G. max* (Sharma *et al.*, 2014; Yonekura-Sakakibara & Hanada, 2011). The combined number of *UGT* genes constitutes approximately 0.61% of the total predicted number of genes in *L. japonicus*; this compares with approximately 0.39%, 0.40%, and 0.52% in *M. truncatula*, *C. arietinum,* and *G. max,* respectively, and is significant for a single class of enzyme in legume species (Sharma *et al.*, 2014). Furthermore, the ratios of *UGT* genes to total genes in these legumes are higher than the respective ratios in *Oryza sativa* (0.32%) and *Zea mays* (0.18%), suggesting that the number of UGTs in legume species expanded more rapidly than in these two graminaceous species.

Our study aimed to systematically characterize the UGT family while integrating phylogenetic and expression pattern data. Most UGTs from *L. japonicus* belonged to 14 major groups (A–N), along with their orthologs from *A. thaliana*. A further five members from group O (UGT93A6–10) did not have orthologs in *A. thaliana*, but this group is present in maize, chickpea, and *Panax ginseng* Meyer (Khorolragchaa *et al.*, 2014; Li *et al.*, 2014; Sharma *et al.*, 2014), and the enzymes of this group are predicted to catalyze the glycosylation of cytokinins (Caputi *et al.*, 2012; Li *et al.*, 2014).

Our analysis revealed that 71 full-length *UGT* genes in *L. japonicus* showed distinctly different expression patterns in various tissues, with many of them being preferentially expressed in a specific tissue (Fig. 2). These global transcript data could be very helpful in the functional identification of the remaining UGT proteins, if combined with metabolic analysis. Such strategies have been successful for the characterization of several *UGT* genes involved in anthocyanin glycosylation in *A. thaliana* (Tohge *et al.*, 2005; Yonekura-Sakakibara *et al.*, 2012).

### Enzymatic characteristics of UGT72 family proteins in *L. japonicus*

Three UGT proteins (UGT72AD1, UGT72AH1, and UGT72Z2) displayed very narrow substrate preferences, with primarily flavonol aglycones as substrates, consistent with their high receptor-ligand score (Supplementary Table S5). In addition, their enzymatic products are primarily 3-*O*-glucosides. These results suggested that these three UGTs have strict substrate specificity and regioselectivity. Similarly, many UGTs that have been characterized to date possess a unique substrate specificity. Some UGTs use flavonols as substrates, such as CsF3GT (Owens & McIntosh, 2009); others, such as GmIF7GT, use isoflavonoids and anthocyanins (Noguchi *et al.*, 2009). UGT proteins that are active toward only one class of flavonoid do not seem to be very common, particularly in the UGT72 family. Similar to UGT72AD1, UGT72AH1, and UGT72Z2, both *VvGT5* and *VvGT6* from *Vitis vinifera* can use flavonols as substrates to produce 3-*O* products. However, *VvGT5* and *VvGT6* can utilize either UDP-glucuronic acid (*VvGT5*) or UDP-glucose/galactose (*VvGT6*) as sugar donors (Ono *et al.*, 2010). Due to their unique substrate preferences and low regioselectivity, UGT72AD1, UGT72AH1, and UGT72Z2 may have potential as biocatalysts for use in the engineering of specific flavonol glucosides. In terms of efficiency, UGT72Z2 may be the most attractive candidate for use as a biocatalyst, as it has a low *K*_m_ relative to UGT72AD1 and UGT72AH1.

UGT72AF1 and UGT72V3 displayed relatively broad substrate preferences, and these proteins can use more than one class of (iso)flavonoids as substrates. Therefore, it was not surprising that these two enzymes had a lower receptor-ligand score than UG72AD1, UG72AH1, or UG72Z2 (Supplementary Table S5). Similarly, several UGTs from other legume species have the same substrates as UG72AF1 and UGT72V3. Four UGT proteins (GT22D, GT22E09, GT29C, and GT29H) in *M. truncatula* displayed activity toward flavonols (kaempferol, myricetin, and/or quercetin), flavones (apigenin and/or lutiolin), and isoflavones (daidzein and genistein) (Modolo *et al.*, 2007). GT04F14 from *P. montana* var. *lobata* and UGT73F2 from *G. max* displayed broad substrate specificity toward isoflavones, flavones, and flavonol aglycones, including daidzein, genistein, quercetin, and apigenin (Dhaubhadel *et al.*, 2008; He *et al.*, 2011). Therefore, UG72AF1 and UGT72V3 appear to be quite typical in terms of their broad substrate preference *in vitro* as compared to UGT proteins of other legume species.

The remaining seven UGTs did not show activity toward flavonoid compounds. Among these, UGT72AD2/UGT72AD1 and UGT72V3/UGT72V2 shared high identity, a close phylogenetic relationship, and a similar expression profile, which suggested that they might have originated via a duplication event. UGT72AD2 and UGT72V2 did not exhibit any activity toward flavonoids, suggesting that their function may have differentiated during evolution.

The five UGTs UGT72AE1, UGT72AG1, UGT72B4, UGT72K1, and UGT72L6 showed relatively high identities with their orthologous proteins from *G. max, P. montana* var. *lobata*, *C. arietinum,* and *P. vulgaris* (Supplementary Table S4), but the UGT proteins from these species either did not show any activities toward flavonoids, such as GT01K01 from *P. montana* var. *lobata* (He *et al.*, 2011), or are only predicted as putative anthocyanidin glucosyltransferases on the basis of sequence similarity. Therefore, these UGT proteins may be redundant genes or they may utilize substrates other than flavonoid aglycones. This is consistent with the suggestion that the function and the substrate specificity of UGT enzymes are not predictable based on sequence analysis alone (Modolo *et al.*, 2007).

### *In vivo* function of *UGT72AD1* and *UGT72Z2*

The enhanced levels of kaempferol 3-*O*-glucoside-7-*O*-rhamnoside and kaempferol 3-*O*-[rhamnosyl(1→2glucoside)]-7-*O*-rhamnoside demonstrated that both UGT72Z2 and UGT72AD1 could accelerate the production of kaempferol 3-*O*-glucoside in *L. japonicus* and *A. thaliana*, consistent with their *in vitro* enzymatic activities. The increased accumulation of kaempferol 3-*O*-rhamnosyl-7-*O*-rhamnoside in the transgenic *A. thaliana* lines suggested that these two UGTs might also be able to utilize UDP-rhamnose as a sugar donor *in vivo*. However, the limited commercial availability of UDP-rhamnose precluded further investigation of this. Among other plant species, due to the diversity of both flavonoids and UGT proteins, the *in vivo* functional characterization of UGTs is generally achieved by loss-of-function in *A. thaliana* (DeBolt *et al.*, 2009; Yonekura-Sakakibara *et al.*, 2012; Yin *et al.*, 2014), but is often lacking in many other plant species (Cui *et al.*, 2016; Dhaubhadel *et al.*, 2008; Falcone Ferreyra *et al.*, 2013; Kannangara *et al.*, 2011; Khater *et al.*, 2012). Therefore, future available mutant resources will perhaps help to establish whether UGT72Z2 and UGT72AD1 are involved in the biosynthesis of flavonol rhamnosides in *L. japonicus*.

UGT72AD1 was highly expressed in leaves and seeds of *L. japonicus*; this was consistent with the pattern of accumulation of kaempferol 3-*O*-glucoside-7-*O*-rhamnoside in these two tissues (Suzuki *et al.*, 2008) and suggests that UGT72AD1 is likely responsible for the biosynthesis of kaempferol 3-*O*-glucoside-7-*O*-rhamnoside in leaves and seeds of *L. japonicus*. The expression of *UGT72AD1* was induced by SA treatment and shared the same trend as the expression pattern of *MYB11* and *MYB14*, suggesting that it may be regulated by MYB11 and MYB14 for the production of flavonol glucosides under adverse conditions. This was the same as for another flavonol glucosyltransferase, *FaGT1*, from strawberry, which was also inducible in fruit by SA treatment (Griesser *et al.*, 2008).

A previous study showed that kaempferol 3-*O*-rhamnoside-7-*O*-rhamnoside acts as an endogenous flavonol inhibitor of polar auxin transport in *A. thaliana* shoots (Yin *et al.*, 2014). In the present study, growth assays revealed that root growth of transgenic *A. thaliana* seedlings was significantly inhibited in two individual lines overexpressing *UGT72AD1* and *UGT72Z2* (*UGT72AD1-19* and *UGT72Z2-18*; Fig. 7). This result is consistent with the increased levels of total flavonol glucosides in these two lines, in particular with the relatively higher levels of kaempferol 3-*O*-rhamnoside-7-*O*-rhamnoside. Therefore, it is possible that the increased levels of kaempferol 3-*O*-rhamnoside-7-*O*-rhamnoside in transgenic *A. thaliana* seedlings overexpressing *UGT72AD1* and *UGT72Z2* inhibited polar auxin transport, which in turn affected root length in these transgenic plants. We speculate that a minor increase in the level of kaempferol 3-*O*-rhamnoside-7-*O*-rhamnoside would lead to only moderate polar auxin transport inhibition, which might not cause obvious phenotypic changes, as seen from the other transgenic lines in the present and previous studies (Noh *et al.*, 2001; Yin *et al.*, 2014). However, the glycosylation pattern of flavonols is quite complex, and there are also several anthocyanidin glycosides and flavonol hexosides present in the flowers and leaves of *L. japonicus* (Suzuki *et al.*, 2008). The biosynthetic and accumulation mechanisms of these glycosylated flavonoid compounds are still unclear, and therefore, questions remain concerning how they are formed in *L. japonicus*. Docking-aided enzymatic assays with the remaining UGTs will almost certainly provide valuable clues to help address these questions.

## Supplementary data

Supplementary data are available at *JXB* online.

Table S1. List of *UGT* genes that were identified in the *L. japonicus* genome.

Table S2. List of full-length *UGT* genes and their corresponding probe set number on the Affymetrix genechip.

Table S3. Sequence identity among 12 members of the UGT72 family in *L. japonicus*.

Table S4. Sequence identity of 12 members of the UGT72 family with their top orthologs.

Table S5. Docking statistic and percentage of substrate converted for the 12 recombinant UGT proteins in *L. japonicus.*

Table S6. Primer sequences used in the present study.

Fig. S1. Genomic distribution of 71 full-length *UTG* genes identified in the *L. japonicus* genome.

Fig. S2. Multiple sequence alignment of 12 members of the UGT72 family in *L. japonicus*.

Fig. S3. Homology docking of 12 members of the UGT72 family in *L. japonicus*.

Fig. S4. Twelve purified recombinant UGT proteins detected on denaturing SDS-PAGE.

Fig. S5. Effects of SA, ABA, and JA treatments on *L. japonicus* hairy roots.

Fig. S6. Overexpression of *UGT72AD1* and *UGT72Z2* genes in hairy roots of *L. japonicus.*

Fig. S7. Representative HPLC chromatograms of *UGT72AD1* and *UGT72Z2* overexpressing lines in hairy roots of *L. japonicus*.

Fig. S8. Representative HPLC chromatograms of *UGT72AD1* and *UGT72Z2* overexpressing lines in seedlings of *A. thaliana*.

Fig. S9. Representative HPLC chromatograms of *UGT72AD1* and *UGT72Z2* overexpression lines in seeds of *A. thaliana*.

## Supplementary Material

supplementary_tables_S1_S6_figures_S1_S9
