# Supplementary material for: Involvement of three putative glucosyltransferases from the UGT72 family in flavonol glucoside/rhamnoside biosynthesis in Lotus japonicus seeds
Source: J Exp Bot. 2016 Nov 18;68(3):597–612. doi: 10.1093/jxb/erw420 (PMC5444469; doi:10.1093/jxb/erw420)
Supplement: supplementary_tables_S1_S6_figures_S1_S9 [file erw420_suppl_supplementary_tables_S1_S6_figures_S1_S9.pdf]

Supplementary Data

Table S1. List of *UGT* genes that were identified in the *L.japonicus* genome

Kazusa identifier	Arabidopsis ortholog	Query length	Hit name	e value	Score	Identity	Positive	Note
chr1.CM0012.2360.r2.d	UGT73C5	311	lcl|UGT73C5 putative glucosyl transferase, chromosome 2	2.00E-125	366	163/288 (57%)	227/288 (79%)	pseudo
chr1.CM0012.2370.r2.a	UGT73C5	486	lcl|UGT73C5 putative glucosyl transferase, chromosome 2	0	589	271/487 (56%)	362/487 (74%)	full-length
chr1.CM0088.150.r2.d	UGT73B3	491	lcl|UGT73B3 AL021961, F28A23	4.00E-173	494	256/488 (52%)	334/488 (68%)	full-length
chr1.CM0105.690.r2.a	UGT72B1	481	lcl|UGT72B1 AL161491, 75572-77014	0	612	298/465 (64%)	356/465 (77%)	full-length
chr1.CM0109.320.r2.m	UGT73B3	471	lcl|UGT73B3 AL021961, F28A23	6.00E-175	498	255/477 (53%)	339/477 (71%)	full-length
chr1.CM0147.400.r2.m	UGT74E1	129	lcl|UGT74E1 AC007153, F3F20, 82737-84239	2.00E-11	53.9	26/38 (68%)	29/38 (76%)	pseudo
chr1.CM0393.1010.r2.m	UGT74F2	212	lcl|UGT74F2 putative glucosyltransferase	5.00E-50	164	89/211 (42%)	124/211 (59%)	partial
chr1.CM0393.1030.r2.m	UGT74E1	238	lcl|UGT74E1 AC007153, F3F20, 82737-84239	5.00E-87	262	127/233 (55%)	165/233 (71%)	partial
chr1.CM0393.1040.r2.m	UGT74F2	217	lcl|UGT74F2 putative glucosyltransferase	5.00E-48	159	87/210 (41%)	123/210 (59%)	partial
chr1.CM0393.1090.r2.d	UGT74D1	268	lcl|UGT74D1 putative glucosyltransferase	5.00E-21	85.1	42/95 (44%)	59/95 (62%)	pseudo
chr1.CM0393.1100.r2.d	UGT74E1	156	lcl|UGT74E1 AC007153, F3F20, 82737-84239	4.00E-43	144	70/119 (59%)	88/119 (74%)	partial
chr1.CM0393.1120.r2.d	UGT74E2	465	lcl|UGT74E2 Similar to indole-3-acetate	7.00E-132	387	205/458 (45%)	286/458 (62%)	full-length
chr1.CM0393.1130.r2.d	UGT74E2	404	lcl|UGT74E2 Similar to indole-3-acetate	5.00E-84	261	123/239 (51%)	169/239 (71%)	pseudo
chr1.CM0393.1250.r2.d	UGT74F2	181	lcl|UGT74F2 putative glucosyltransferase	4.00E-37	128	69/159 (43%)	97/159 (61%)	partial
chr1.CM0393.980.r2.m	UGT74E2	457	lcl|UGT74E2 Similar to indole-3-acetate	2.00E-127	375	203/457 (44%)	276/457 (60%)	full-length
chr1.CM0393.990.r2.m	UGT74C1	277	lcl|UGT74C1 putative glucosyltransferase	3.00E-46	157	71/129 (55%)	92/129 (71%)	pseudo
chr1.CM0410.100.r2.m	UGT83A1	440	lcl|UGT83A1 similar to UDP-glucose glucosyltransferase	3.00E-114	341	186/463 (40%)	277/463 (60%)	full-length
chr1.CM0410.110.r2.m	UGT83A1	445	lcl|UGT83A1 similar to UDP-glucose glucosyltransferase	1.00E-130	383	195/460 (42%)	282/460 (61%)	full-length
chr1.CM0410.130.r2.m	UGT83A1	445	lcl|UGT83A1 similar to UDP-glucose glucosyltransferase	2.00E-123	364	187/460 (41%)	277/460 (60%)	full-length
chr1.CM0410.140.r2.m	UGT83A1	446	lcl|UGT83A1 similar to UDP-glucose glucosyltransferase	1.00E-119	355	192/462 (42%)	281/462 (61%)	full-length
chr1.CM0410.150.r2.m	UGT83A1	448	lcl|UGT83A1 similar to UDP-glucose glucosyltransferase	5.00E-133	389	199/461 (43%)	289/461 (63%)	full-length

chr1.CM0591.560.r2.d	UGT73B3	507	lcl|UGT73B3 AL021961, F28A23	4.00E-162	466	239/504 (47%)	330/504 (65%)	full-length
chr1.CM0955.210.r2.d	UGT81A3	36	lcl|UGT81A3 AJ000331, monogalactosyldiacylglycerol synthase	7.00E-09	43.1	17/23 (74%)	19/23 (83%)	pseudo
chr1.CM1413.240.r2.a	UGT80A2	505	lcl|UGT80A2 Z83833, UDP-glucose:sterol glucosyltransferase	0	750	363/510 (71%)	414/510 (81%)	partial
chr1.CM1868.170.r2.m	UGT89B1	211	lcl|UGT89B1 AC016662, F2P9, 86614-88035	2.00E-36	127	67/122 (55%)	87/122 (71%)	pseudo
chr1.CM1868.190.r2.m	UGT89B1	480	lcl|UGT89B1 AC016662, F2P9, 86614-88035	1.00E-178	507	261/468 (56%)	328/468 (70%)	full-length
chr1.LjT10H09.40.r2.m	UGT72B1	482	lcl|UGT72B1 AL161491, 75572-77014	6.00E-136	399	210/489 (43%)	304/489 (62%)	full-length
chr2.CM0028.830.r2.d	UGT74B1	221	lcl|UGT74B1 A. thaliana, Similar to glucosyltransferases, (S-thiohydroximate-glucosyltransferrase	6.00E-89	267	126/224 (56%)	175/224 (78%)	partial
chr2.CM0102.50.r2.d	UGT82A1	78	lcl|UGT82A1 AP002046, MMP21, 6263-8510	2.00E-19	75.1	37/76 (49%)	50/76 (66%)	partial
chr2.CM0102.70.r2.d	UGT82A1	405	lcl|UGT82A1 AP002046, MMP21, 6263-8510	8.00E-138	400	194/360 (54%)	264/360 (73%)	partial
chr2.CM0124.1460.r2.m	UGT89A2	461	lcl|UGT89A2 AL162751, 85361-86758	3.00E-159	457	227/453 (50%)	309/453 (68%)	full-length
chr2.CM0124.440.r2.m	UGT85A2	481	lcl|UGT85A2 chromosome 1	0	575	265/484 (55%)	367/484 (76%)	full-length
chr2.CM0533.380.r2.a	UGT88A1	457	lcl|UGT88A1 flavonol 3-O-glucosyltransferase-like protein	3.00E-55	186	114/344 (33%)	180/344 (52%)	full-length
chr2.CM1882.140.r2.a	UGT85A6P	268	lcl|UGT85A6P AC068562, T16E15.4, 6537-8157	2.00E-45	154	69/129 (53%)	93/129 (72%)	pseudo
chr2.CM1882.160.r2.a	UGT85A2	630	lcl|UGT85A2 chromosome 1	0	576	272/475 (57%)	353/475 (74%)	partial
chr2.CM1882.260.r2.a	UGT83A1	454	lcl|UGT83A1 similar to UDP-glucose glucosyltransferase	9.00E-172	488	249/464 (54%)	318/464 (69%)	full-length
chr2.CM1882.280.r2.a	UGT83A1	463	lcl|UGT83A1 similar to UDP-glucose glucosyltransferase	6.00E-123	364	198/471 (42%)	283/471 (60%)	full-length
chr2.LjT02C03.170.r2.d	UGT91B1	232	lcl|UGT91B1 anthocyanidin-3-glucoside rhamnosyltransferase	8.00E-50	165	101/239 (42%)	136/239 (57%)	full-length
chr2.LjT09A09.10.r2.d	UGT87A1	457	lcl|UGT87A1 AC004165, T27E13, 50431-51895	5.00E-152	438	225/451 (50%)	299/451 (66%)	pseudo
chr2.LjT09A09.20.r2.d	UGT87A1	447	lcl|UGT87A1 AC004165, T27E13, 50431-51895	9.00E-154	442	220/449 (49%)	304/449 (68%)	pseudo
chr2.LjT09A09.30.r2.d	UGT87A1	474	lcl|UGT87A1 AC004165, T27E13, 50431-51895	7.00E-50	172	81/171 (47%)	119/171 (70%)	pseudo
chr2.LjT09A09.80.r2.d	UGT87A1	488	lcl|UGT87A1 AC004165, T27E13, 50431-51895	1.00E-160	461	232/486 (48%)	321/486 (66%)	full-length
chr3.CM0135.160.r2.d	UGT72B1	516	lcl|UGT72B1 AL161491, 75572-77014	6.00E-126	374	204/479 (43%)	285/479 (59%)	full-length
chr3.CM0241.610.r2.m	UGT85A7	480	lcl|UGT85A7 AC068562, T16E15.5, 8945-10571	0	540	244/475 (51%)	336/475 (71%)	full-length
chr3.CM0282.130.r2.d	UGT79B10	174	lcl|UGT79B10 38% identical to Flavonol 3-O-Glucosyltransferase	2.00E-42	142	66/108 (61%)	84/108 (78%)	pseudo
chr3.CM0282.30.r2.d	UGT79B3	191	lcl|UGT79B3 strong similarity to UDP rhamnose--anthocyanidin-3-glucoside	2.00E-29	107	63/166 (38%)	87/166 (52%)	pseudo

chr3.CM0282.40.r2.d	UGT79B1	208	lcl|UGT79B1 flavonol 3-O-glucosyltransferase-like	2.00E-47	157	80/181 (44%)	114/181 (63%)	pseudo
chr3.CM0282.590.r2.m	UGT79B6	485	lcl|UGT79B6 AB007644, K19P17, 59557-60918	1.00E-97	299	144/305 (47%)	199/305 (65%)	pseudo
chr3.CM0282.950.r2.m	UGT72D2P	474	lcl|UGT72D2P AC006135, F24H14, 55344-56752	3.00E-154	445	230/474 (49%)	314/474 (66%)	full-length
chr3.CM0282.980.r2.m	UGT72D2P	470	lcl|UGT72D2P AC006135, F24H14, 55344-56752	2.00E-159	458	244/475 (51%)	321/475 (68%)	full-length
chr3.CM0574.280.r2.m	UGT71C4	259	lcl|UGT71C4 AC067971, F10K1.4, 14158-15597	2.00E-13	62.8	64/221 (29%)	96/221 (43%)	pseudo
chr3.CM0574.90.r2.d	UGT72B1	473	lcl|UGT72B1 AL161491, 75572-77014	5.00E-170	486	243/470 (52%)	316/470 (67%)	full-length
chr3.CM0619.120.r2.d	UGT78D1	156	lcl|UGT78D1 Contains similarity to gb|AF000372 UDP glucose:flavanoid	1.00E-11	55.1	28/85 (33%)	47/85 (55%)	partial
chr3.CM0797.150.r2.d	UGT73B5	384	lcl|UGT73B5 putative glucosyltransferase	3.00E-74	236	117/340 (34%)	190/340 (56%)	pseudo
chr3.CM0846.120.r2.d	UGT72B1	474	lcl|UGT72B1 AL161491, 75572-77014	4.00E-148	429	214/472 (45%)	318/472 (67%)	full-length
chr3.CM0846.130.r2.d	UGT72B1	475	lcl|UGT72B1 AL161491, 75572-77014	5.00E-84	264	122/215 (57%)	160/215 (74%)	pseudo
chr3.LjB09K03.30.r2.m	UGT92A1	484	lcl|UGT92A1 AL353013, T24H18, 21475-22941	0	525	256/485 (53%)	340/485 (70%)	full-length
chr3.LjT14J20.130.r2.m	UGT79B11	138	lcl|UGT79B11 37% identical to Flavonol 3-O-Glucosyltransferase	1.00E-31	111	47/81 (58%)	63/81 (78%)	pseudo
chr3.LjT14J20.160.r2.m	UGT79B6	459	lcl|UGT79B6 AB007644, K19P17, 59557-60918	8.00E-161	460	222/456 (49%)	305/456 (67%)	full-length
chr3.LjT14J20.170.r2.m	UGT79B6	469	lcl|UGT79B6 AB007644, K19P17, 59557-60918	7.00E-150	433	214/456 (47%)	297/456 (65%)	full-length
chr3.LjT24D06.90.r2.d	UGT85A2	252	lcl|UGT85A2 chromosome 1	9.00E-77	237	115/168 (68%)	139/168 (83%)	partial
chr3.LjT35O23.150.r2.d	UGT72D1	487	lcl|UGT72D1 putative flavonol 3-O-glucosyltransferase	1.00E-130	385	202/479 (42%)	307/479 (64%)	full-length
chr3.LjT41F16.10.r2.d	UGT72B3	245	lcl|UGT72B3 AC023628, F6F3.22, 95474-96919	1.00E-74	231	118/241 (49%)	165/241 (68%)	partial
chr3.LjT41F16.40.r2.d	UGT72B1	260	lcl|UGT72B1 AL161491, 75572-77014	2.00E-93	280	130/210 (62%)	172/210 (82%)	partial
chr3.LjT45I18.30.r2.d	UGT85A7	481	lcl|UGT85A7 AC068562, T16E15.5, 8945-10571	0	533	242/478 (51%)	335/478 (70%)	full-length
chr4.CM0042.1820.r2.m	UGT81A4	457	lcl|UGT81A4 AB047398, MGDG synthase type C	1.00E-06	43.1	38/120 (32%)	56/120 (47%)	pseudo
chr4.CM0044.480.r2.m	UGT75D1	446	lcl|UGT75D1 transfers the glucose moeity from UDP-glucose	7.00E-119	353	197/464 (42%)	274/464 (59%)	pseudo
chr4.CM0044.490.r2.m	UGT75B1	457	lcl|UGT75B1 hypothetical UDP-glucose:anthocyanin	2.00E-141	411	223/465 (48%)	298/465 (64%)	full-length
chr4.CM0175.320.r2.m	UGT81A4	73	lcl|UGT81A4 AB047398, MGDG synthase type C	4.00E-18	71.2	36/68 (53%)	47/68 (69%)	partial
chr4.CM0219.90.r2.d	UGT81A3	462	lcl|UGT81A3 AJ000331, monogalactosyldiacylglycerol synthase	0	674	345/469 (74%)	391/469 (83%)	full-length
chr4.CM0227.640.r2.m	UGT73B4	474	lcl|UGT73B4 AC006248, F26H6, 11756-13404	2.00E-141	412	214/486 (44%)	300/486 (62%)	full-length
chr4.CM0227.650.r2.m	UGT73B4	472	lcl|UGT73B4 AC006248, F26H6, 11756-13404	4.00E-176	501	247/482 (51%)	330/482 (68%)	full-length

chr4.CM0227.660.r2.m	UGT73B3	485	lcl|UGT73B3 AL021961, F28A23	0	514	256/481 (53%)	340/481 (71%)	full-length
chr4.CM0227.690.r2.m	UGT73B3	486	lcl|UGT73B3 AL021961, F28A23	0	539	274/489 (56%)	351/489 (72%)	full-length
chr4.CM0387.270.r2.d	UGT73B4	472	lcl|UGT73B4 AC006248, F26H6, 11756-13404	8.00E-129	380	205/484 (42%)	288/484 (60%)	full-length
chr4.CM0387.340.r2.d	UGT73B4	507	lcl|UGT73B4 AC006248, F26H6, 11756-13404	3.00E-131	387	208/482 (43%)	295/482 (61%)	full-length
chr4.CM0387.360.r2.d	UGT73B4	466	lcl|UGT73B4 AC006248, F26H6, 11756-13404	2.00E-124	369	201/484 (42%)	283/484 (58%)	full-length
chr4.CM0387.370.r2.d	UGT73B4	481	lcl|UGT73B4 AC006248, F26H6, 11756-13404	8.00E-131	385	209/491 (43%)	289/491 (59%)	full-length
chr4.CM0387.390.r2.d	UGT73B4	494	lcl|UGT73B4 AC006248, F26H6, 11756-13404	2.00E-137	403	214/488 (44%)	303/488 (62%)	full-length
chr4.CM0414.390.r2.d	UGT85A2	177	lcl|UGT85A2 chromosome 1	3.00E-67	209	92/164 (56%)	117/164 (71%)	partial
chr4.CM0528.110.r2.d	UGT84B2	465	lcl|UGT84B2 AC002391, F21P24, 56464-57816	1.00E-140	409	207/458 (45%)	296/458 (65%)	full-length
chr5.CM0034.480.r2.d	UGT73C7	116	lcl|UGT73C7 strong similarity to glucosyltransferase IS5a,	9.00E-09	45.4	19/48 (40%)	33/48 (69%)	partial
chr5.CM0040.760.r2.m	UGT81A3	53	lcl|UGT81A3 AJ000331, monogalactosyldiacylglycerol synthase	9.00E-11	49.3	18/23 (78%)	20/23 (87%)	pseudo
chr5.CM0052.810.r2.m	UGT80B1	638	lcl|UGT80B1 NM_103487, GI:18400961	0	879	437/620 (70%)	512/620 (83%)	full-length
chr5.CM0200.3220.r2.m	UGT84A1	479	lcl|UGT84A1 similarity to indole-3-acetate	0	519	244/468 (52%)	343/468 (73%)	full-length
chr5.CM0200.3230.r2.m	UGT84B1	490	lcl|UGT84B1 putative glucosyltransferase	1.00E-136	400	213/481 (44%)	304/481 (63%)	full-length
chr5.CM0278.660.r2.m	UGT73C3	477	lcl|UGT73C3 putative glucosyltransferase	3.00E-60	201	148/498 (30%)	241/498 (48%)	full-length
chr5.CM1077.490.r2.m	UGT73C1	484	lcl|UGT73C1 putative glucosyltransferase	3.00E-112	338	175/350 (50%)	230/350 (66%)	pseudo
chr5.CM1077.550.r2.m	UGT73C3	497	lcl|UGT73C3 putative glucosyltransferase	5.00E-156	451	229/498 (46%)	338/498 (68%)	full-length
chr5.CM1077.560.r2.m	UGT73D1	234	lcl|UGT73D1 strong similarity to glucosyltransferase IS10a,	2.00E-64	205	100/232 (43%)	157/232 (68%)	partial
chr5.CM1077.590.r2.m	UGT73C2	484	lcl|UGT73C2 putative glucosyltransferase	0	542	265/489 (54%)	346/489 (71%)	full-length
chr5.CM1077.600.r2.m	UGT73C3	498	lcl|UGT73C3 putative glucosyltransferase	6.00E-156	451	226/496 (46%)	336/496 (68%)	full-length
chr5.CM1077.610.r2.m	UGT73C1	499	lcl|UGT73C1 putative glucosyltransferase	5.00E-174	497	241/486 (50%)	344/486 (71%)	full-length
chr5.CM1077.630.r2.m	UGT73D1	487	lcl|UGT73D1 strong similarity to glucosyltransferase IS10a,	0	619	295/493 (60%)	387/493 (78%)	full-length
chr5.CM2155.140.r2.a	UGT72B3	478	lcl|UGT72B3 AC023628, F6F3.22, 95474-96919	6.00E-124	368	208/488 (43%)	302/488 (62%)	full-length
chr6.CM0055.510.r2.m	UGT78D2	454	lcl|UGT78D2 AL391141, F2K13, 64922-66486	9.00E-144	417	204/450 (45%)	294/450 (65%)	full-length
chr6.CM0114.250.r2.m	UGT79B6	456	lcl|UGT79B6 AB007644, K19P17, 59557-60918	1.00E-164	470	219/454 (48%)	311/454 (69%)	full-length
chr6.CM0118.1240.r2.d	UGT72E1	469	lcl|UGT72E1 Contains UDP-glycosyltransferases signature	4.00E-114	342	187/464 (40%)	283/464 (61%)	full-length

chr6.CM0118.1250.r2.d	UGT72E1	465	lcl|UGT72E1 Contains UDP-glycosyltransferases signature	6.00E-82	258	143/345 (41%)	213/345 (62%)	pseudo
chr6.CM0314.440.r2.m	UGT91A1	477	lcl|UGT91A1 putative anthocyanidin-3-glucoside	1.00E-130	384	194/474 (41%)	293/474 (62%)	full-length
chr6.CM0314.450.r2.m	UGT91A1	476	lcl|UGT91A1 putative anthocyanidin-3-glucoside	3.00E-127	375	195/468 (42%)	287/468 (61%)	full-length
chr6.CM0314.490.r2.m	UGT91A1	490	lcl|UGT91A1 putative anthocyanidin-3-glucoside	7.00E-139	406	212/477 (44%)	292/477 (61%)	full-length
chr6.CM0314.500.r2.d	UGT85A2	482	lcl|UGT85A2 chromosome 1	0	567	259/476 (54%)	357/476 (75%)	pseudo
chr6.CM0367.140.r2.m	UGT73C3	481	lcl|UGT73C3 putative glucosyltransferase	6.00E-108	327	184/490 (38%)	272/490 (56%)	full-length
chr6.CM1757.170.r2.a	UGT81A4	57	lcl|UGT81A4 AB047398, MGDG synthase type C	6.00E-25	89.7	40/57 (70%)	45/57 (79%)	pseudo
chr6.LjT14E02.150.r2.d	UGT76F2	460	lcl|UGT76F2 AL161667, F1I16, 59290-61366	2.00E-172	490	249/469 (53%)	328/469 (70%)	full-length
LjSGA_003166.2	UGT85A2	484	lcl|UGT85A2 chromosome 1	0	665	318/485 (66%)	394/485 (81%)	full-length
LjSGA_004982.1	UGT79B3	262	lcl|UGT79B3 strong similarity to UDP rhamnose--anthocyanidin-3-glucoside	5.00E-109	320	158/255 (62%)	188/255 (74%)	partial
LjSGA_013199.1	UGT71B6	138	lcl|UGT71B6 AB025634, MSD21, 33372-31939	2.00E-15	65.9	47/136 (35%)	70/136 (51%)	pseudo
LjSGA_014618.2	UGT73B4	228	lcl|UGT73B4 AC006248, F26H6, 11756-13404	5.00E-58	187	108/217 (50%)	137/217 (63%)	partial
LjSGA_015360.1	UGT74F1	459	lcl|UGT74F1 putative glucosyltransferase	6.00E-149	430	208/450 (46%)	305/450 (68%)	full-length
LjSGA_015919.1	UGT72E1	508	lcl|UGT72E1 Contains UDP-glycosyltransferases signature	9.00E-159	458	238/473 (50%)	324/473 (68%)	full-length
LjSGA_016571.1	UGT72B1	489	lcl|UGT72B1 AL161491, 75572-77014	4.00E-160	461	233/476 (49%)	313/476 (66%)	full-length
LjSGA_021337.2	UGT80B1	147	lcl|UGT80B1 NM_103487, GI:18400961	9.00E-65	204	101/136 (74%)	117/136 (86%)	pseudo
LjSGA_021883.1	UGT71C3	177	lcl|UGT71C3 AC067971, F10K1.3, 12382-13812	6.00E-68	211	101/175 (58%)	133/175 (76%)	partial
LjSGA_023327.1	UGT85A3	153	lcl|UGT85A3 AC006551, F12K8, 105703-107198; AC068562, T16E15, 1-513	2.00E-21	83.6	54/154 (35%)	73/154 (47%)	partial
LjSGA_024674.1	UGT80B1	102	lcl|UGT80B1 NM_103487, GI:18400961	2.00E-24	90.9	37/45 (82%)	41/45 (91%)	pseudo
LjSGA_025093.1	UGT72B1	270	lcl|UGT72B1 AL161491, 75572-77014	9.00E-105	310	149/269 (55%)	198/269 (74%)	partial
LjSGA_027597.1	UGT73B1	472	lcl|UGT73B1 AL021961, F28A23	2.00E-106	323	195/485 (40%)	280/485 (58%)	partial
LjSGA_028050.1	UGT91A1	473	lcl|UGT91A1 putative anthocyanidin-3-glucoside	3.00E-130	383	206/459 (45%)	280/459 (61%)	full-length
LjSGA_028537.1	UGT85A2	308	lcl|UGT85A2 chromosome 1	7.00E-73	229	122/301 (41%)	184/301 (61%)	partial
LjSGA_029106.1	UGT79B6	414	lcl|UGT79B6 AB007644, K19P17, 59557-60918	6.00E-134	390	187/410 (46%)	271/410 (66%)	partial
LjSGA_029279.2	UGT79B1	307	lcl|UGT79B1 flavonol 3-O-glucosyltransferase-like	6.00E-97	291	147/316 (47%)	206/316 (65%)	pseudo
LjSGA_030028.1	UGT80A2	142	lcl|UGT80A2 Z83833, UDP-glucose:sterol glucosyltransferase	5.00E-59	189	84/111 (76%)	96/111 (86%)	partial

LjSGA_032065.1	UGT72D2P	274	lcl|UGT72D2P AC006135, F24H14, 55344-56752	1.00E-41	144	70/136 (51%)	96/136 (71%)	pseudo
LjSGA_032783.2	UGT87A1	188	lcl|UGT87A1 AC004165, T27E13, 50431-51895	7.00E-40	136	69/148 (47%)	94/148 (64%)	partial
LjSGA_033686.1	UGT73B3	242	lcl|UGT73B3 AL021961, F28A23	4.00E-51	169	89/246 (36%)	141/246 (57%)	partial
LjSGA_037880.1	UGT85A5	183	lcl|UGT85A5 AC068562, T16E15.2, 1156-2919	2.00E-68	213	86/168 (51%)	126/168 (75%)	partial
LjSGA_040125.1	UGT81A1	200	lcl|UGT81A1 monogalactosyldiacylglycerol synthase - like protein	2.00E-103	305	142/186 (76%)	158/186 (85%)	pseudo
LjSGA_040150.1	UGT78D2	180	lcl|UGT78D2 AL391141, F2K13, 64922-66486	2.00E-53	172	80/159 (50%)	111/159 (70%)	partial
LjSGA_042817.1	UGT73C4	437	lcl|UGT73C4 putative glucosyltransferase	3.00E-58	195	142/450 (32%)	214/450 (48%)	partial
LjSGA_043170.1	UGT78D1	32	lcl|UGT78D1 Contains similarity to gb|AF000372 UDP glucose:flavanoid	4.00E-06	34.7	17/26 (65%)	21/26 (81%)	partial
LjSGA_044016.1	UGT85A2	152	lcl|UGT85A2 chromosome 1	2.00E-59	188	84/149 (56%)	109/149 (73%)	partial
LjSGA_044094.1	UGT85A6P	168	lcl|UGT85A6P AC068562, T16E15.4, 6537-8157	5.00E-69	214	98/169 (58%)	130/169 (77%)	partial
LjSGA_045743.1	UGT88A1	145	lcl|UGT88A1 flavonol 3-O-glucosyltransferase-like protein	1.00E-20	81.3	41/119 (34%)	62/119 (52%)	partial
LjSGA_045779.1.1	UGT78D2	104	lcl|UGT78D2 AL391141, F2K13, 64922-66486	4.00E-21	80.9	42/101 (42%)	62/101 (61%)	partial
LjSGA_046111.1	UGT73B3	272	lcl|UGT73B3 AL021961, F28A23	2.00E-51	171	96/271 (35%)	151/271 (56%)	partial
LjSGA_046960.1	UGT79B1	265	lcl|UGT79B1 flavonol 3-O-glucosyltransferase-like	3.00E-75	233	118/269 (44%)	167/269 (62%)	partial
LjSGA_047409.1	UGT85A2	75	lcl|UGT85A2 chromosome 1	4.00E-30	104	44/65 (68%)	53/65 (82%)	partial
LjSGA_047474.1	UGT91C1	237	lcl|UGT91C1 anthocyanidin-3-glucoside	5.00E-79	242	113/233 (48%)	156/233 (67%)	partial
LjSGA_047571.1	UGT72B3	286	lcl|UGT72B3 AC023628, F6F3.22, 95474-96919	9.00E-60	194	120/286 (42%)	170/286 (59%)	partial
LjSGA_047870.1	UGT73C3	166	lcl|UGT73C3 putative glucosyltransferase	1.00E-34	120	75/176 (43%)	98/176 (56%)	partial
LjSGA_048307.1	UGT71B1	259	lcl|UGT71B1 AB025634, MSD21, 21447-22865	8.00E-30	110	80/259 (31%)	134/259 (52%)	partial
LjSGA_048844.2.1	UGT92A1	164	lcl|UGT92A1 AL353013, T24H18, 21475-22941	6.00E-15	65.1	32/57 (56%)	45/57 (79%)	pseudo
LjSGA_049701.1	UGT80A2	127	lcl|UGT80A2 Z83833, UDP-glucose:sterol glucosyltransferase	6.00E-45	149	74/106 (70%)	80/106 (75%)	pseudo
LjSGA_050341.1	UGT71D2	190	lcl|UGT71D2 AC005496, T27A16, 40783-42186	3.00E-38	131	72/192 (38%)	113/192 (59%)	partial
LjSGA_052002.1	UGT73C3	334	lcl|UGT73C3 putative glucosyltransferase	2.00E-103	310	156/335 (47%)	224/335 (67%)	partial
LjSGA_052966.2	UGT72C1	156	lcl|UGT72C1 strong similarity to UDP-glucose	5.00E-33	115	55/116 (47%)	77/116 (66%)	pseudo
LjSGA_053314.2	UGT72B3	194	lcl|UGT72B3 AC023628, F6F3.22, 95474-96919	1.00E-50	166	86/193 (45%)	117/193 (61%)	partial
LjSGA_060963.2	UGT79B3	143	lcl|UGT79B3 strong similarity to UDP rhamnose--anthocyanidin-3-glucoside	1.00E-46	152	65/135 (48%)	103/135 (76%)	pseudo

LjSGA_063463.1	UGT80B1	61	lcl|UGT80B1 NM_103487, GI:18400961	2.00E-24	89.4	41/64 (64%)	49/64 (77%)	partial
LjSGA_065630.1	UGT73B2	194	lcl|UGT73B2 AL021961, F28A23	3.00E-47	157	76/192 (40%)	111/192 (58%)	partial
LjSGA_066071.1	UGT85A2	201	lcl|UGT85A2 chromosome 1	8.00E-71	219	97/162 (60%)	123/162 (76%)	pseudo
LjSGA_066183.1	UGT88A1	301	lcl|UGT88A1 flavonol 3-O-glucosyltransferase-like protein	3.00E-97	291	154/298 (52%)	202/298 (68%)	partial
LjSGA_068283.2	UGT89A1P	341	lcl|UGT89A1P AAD30635-1, possibly a pseudogene (gene is about 30 aa too short).	1.00E-07	45.8	26/86 (30%)	45/86 (52%)	pseudo
LjSGA_071709.1	UGT79B3	281	lcl|UGT79B3 strong similarity to UDP rhamnose--anthocyanidin-3-glucoside	3.00E-96	288	146/278 (53%)	185/278 (67%)	partial
LjSGA_071841.2	UGT73B5	408	lcl|UGT73B5 putative glucosyltransferase	4.00E-103	311	170/414 (41%)	248/414 (60%)	partial
LjSGA_076799.1	UGT71B2	266	lcl|UGT71B2 AB025634, MSD21, 23981-25438	2.00E-92	278	140/261 (54%)	184/261 (70%)	pseudo
LjSGA_077173.1	UGT81A1	178	lcl|UGT81A1 monogalactosyldiacylglycerol synthase - like protein	4.00E-34	120	85/187 (45%)	106/187 (57%)	partial
LjSGA_083752.1	UGT81A1	38	lcl|UGT81A1 monogalactosyldiacylglycerol synthase - like protein	2.00E-23	84.7	37/38 (97%)	37/38 (97%)	partial
LjSGA_084705.1	UGT71C3	266	lcl|UGT71C3 AC067971, F10K1.3, 12382-13812	2.00E-94	283	133/265 (50%)	188/265 (71%)	partial
LjSGA_086482.1	UGT74F1	311	lcl|UGT74F1 putative glucosyltransferase	1.00E-95	287	144/303 (48%)	205/303 (68%)	partial
LjSGA_091755.2	UGT85A7	231	lcl|UGT85A7 AC068562, T16E15.5, 8945-10571	7.00E-91	273	125/215 (58%)	170/215 (79%)	partial
LjSGA_092302.1	UGT73C3	271	lcl|UGT73C3 putative glucosyltransferase	1.00E-73	230	118/250 (47%)	164/250 (66%)	partial
LjSGA_095026.1	UGT72E1	359	lcl|UGT72E1 Contains UDP-glycosyltransferases signature	1.00E-77	243	123/249 (49%)	166/249 (67%)	pseudo
LjSGA_096934.1	UGT79B6	304	lcl|UGT79B6 AB007644, K19P17, 59557-60918	8.00E-95	285	145/306 (47%)	205/306 (67%)	partial
LjSGA_098269.1	UGT71D1	185	lcl|UGT71D1 AC005496, T27A16, 45718-47121	1.00E-36	127	70/184 (38%)	114/184 (62%)	partial
LjSGA_102534.1	UGT85A5	154	lcl|UGT85A5 AC068562, T16E15.2, 1156-2919	5.00E-55	176	82/146 (56%)	106/146 (73%)	partial
LjSGA_104176.1	UGT88A1	205	lcl|UGT88A1 flavonol 3-O-glucosyltransferase-like protein	2.00E-73	226	112/205 (55%)	148/205 (72%)	partial
LjSGA_108549.1	UGT71D1	147	lcl|UGT71D1 AC005496, T27A16, 45718-47121	4.00E-30	107	56/135 (41%)	87/135 (64%)	partial
LjSGA_114591.1	UGT88A1	215	lcl|UGT88A1 flavonol 3-O-glucosyltransferase-like protein	5.00E-70	218	109/203 (54%)	146/203 (72%)	pseudo
LjSGA_126577.1	UGT71C3	295	lcl|UGT71C3 AC067971, F10K1.3, 12382-13812	3.00E-98	294	141/296 (48%)	201/296 (68%)	partial
LjSGA_128268.1	UGT72B1	223	lcl|UGT72B1 AL161491, 75572-77014	1.00E-92	277	131/225 (58%)	175/225 (78%)	partial
LjSGA_146123.1.1	UGT88A1	208	lcl|UGT88A1 flavonol 3-O-glucosyltransferase-like protein	6.00E-30	108	78/202 (39%)	113/202 (56%)	pseudo
LjSGA_148423.1	UGT72B1	171	lcl|UGT72B1 AL161491, 75572-77014	6.00E-45	149	82/169 (49%)	111/169 (66%)	partial
LjSGA_150111.1	UGT71B7	184	lcl|UGT71B7 AB025634, MSD21, 33809-35296	3.00E-33	117	56/106 (53%)	75/106 (71%)	pseudo

LjSGA_150780.1	UGT87A2	51	lcl|UGT87A2 putative glucosyltransferase	1.00E-07	40.4	20/44 (45%)	29/44 (66%)	partial
LjT01K14.20.r2.d	UGT74F1	458	lcl|UGT74F1 putative glucosyltransferase	5.00E-150	432	218/448 (49%)	307/448 (69%)	full-length
LjT06A15.20.r2.d	UGT87A2	61	lcl|UGT87A2 putative glucosyltransferase	5.00E-09	44.7	24/77 (31%)	39/77 (51%)	partial
LjT09A12.10.r2.m	UGT73B3	474	lcl|UGT73B3 AL021961, F28A23	6.00E-62	205	144/487 (30%)	238/487 (49%)	full-length
LjT09A12.20.r2.m	UGT73B3	475	lcl|UGT73B3 AL021961, F28A23	2.00E-58	196	141/486 (29%)	234/486 (48%)	full-length
LjT09A12.30.r2.m	UGT72C1	177	lcl|UGT72C1 strong similarity to UDP-glucose	1.00E-41	140	73/166 (44%)	105/166 (63%)	partial
LjT09A12.60.r2.m	UGT73B4	477	lcl|UGT73B4 AC006248, F26H6, 11756-13404	2.00E-53	182	135/491 (27%)	232/491 (47%)	full-length
LjT09A12.80.r2.m	UGT73B3	473	lcl|UGT73B3 AL021961, F28A23	4.00E-56	189	147/484 (30%)	230/484 (48%)	full-length
LjT38K16.60.r2.a	UGT73B4	405	lcl|UGT73B4 AC006248, F26H6, 11756-13404	2.00E-59	197	106/229 (46%)	143/229 (62%)	pseudo
LjT44J06.210.r2.d	UGT89B1	434	lcl|UGT89B1 AC016662, F2P9, 86614-88035	2.00E-99	303	178/406 (44%)	241/406 (59%)	pseudo
LjT46F11.10.r2.a	UGT90A1	399	lcl|UGT90A1 AC005167, F12A24, 24146-26230	5.00E-148	426	218/407 (54%)	281/407 (69%)	partial
LjT48O01.70.r2.d	UGT81A4	86	lcl|UGT81A4 AB047398, MGDG synthase type C	2.00E-25	92	37/59 (63%)	46/59 (78%)	pseudo

Table S2 List of full-length *UGT* genes and their corresponding probe set number on the Affymetrix genechip

Kazusa identifer	Name	Probe set number	e value
chr1.CM0012.2370.r2.a	UGT73C17	chr1.CM0012.80_at	4.00E-23
chr2.CM0533.380.r2.a	UGT708D2	Ljwgs_022630.3_at	0
chr1.LjT10H09.40.r2.m	UGT72AD1	chr1.TM0189.6_s_at	0
chr3.CM0135.160.r2.d	UGT72AD2	chr3.CM0135.10_at	0
chr5.CM2155.140.r2.a	UGT72AE1	Ljwgs_011402.3_at	0
chr6.CM0118.1240.r2.d	UGT72AF1	TM0674.25_at	0
LjSGA_015919.1	UGT72AG1	Ljwgs_015919.1_at	0
chr3.CM0846.120.r2.d	UGT72AH1	chr3.TM0984.8_at	0
chr1.CM0105.690.r2.a	UGT72B4	chr1.CM0579.4_at	0
chr5.CM0200.3220.r2.m	UGT84F5	chr5.CM0200.56_at	0
chr3.LjT35O23.150.r2.d	UGT72K1	chr3.TM1465.1_at	0
chr3.CM0574.90.r2.d	UGT72L6	chr3.CM0574.27_at	0
chr3.CM0282.980.r2.m	UGT72V2	chr3.CM0282.132_at	0
chr3.CM0282.950.r2.m	UGT72V3	chr3.CM0282.124_at	0
LjSGA_016571.1	UGT72Z2	Ljwgs_016571.1_at	0
chr5.CM1077.610.r2.m	UGT73AA2	Ljwgs_092302.1_at	1.00E-16
chr5.CM1077.630.r2.m	UGT73AB2	chr1.CM0012.80_at	3.00E-08
chr4.CM0227.640.r2.m	UGT73AC2	Ljwgs_009735.1_at	0
chr5.CM1077.550.r2.m	UGT73AJ1	chr1.CM0012.80_at	5.00E-07
chr5.CM1077.600.r2.m	UGT73AJ2	chr1.CM0012.80_at	8.00E-12
chr6.CM0367.140.r2.m	UGT73AK1	Ljwgs_135723.1_s_at	0
chr1.CM0088.150.r2.d	UGT73B17	chr1.CM0088.14_at	0
chr1.CM0591.560.r2.d	UGT73B18	chr1.CM0591.45.1_at	0
chr1.CM0109.320.r2.m	UGT73B19	chr1.CM0109.36_at	0

chr4.CM0227.690.r2.m	UGT73B20	Ljwgs_012092.1_at	0
chr4.CM0227.660.r2.m	UGT73B21	Ljwgs_012092.1_at	1.00E-44
chr4.CM0227.650.r2.m	UGT73B22	Ljwgs_012092.1_at	8.00E-15
chr5.CM1077.590.r2.m	UGT73C18	Ljwgs_053613.2_at	0
chr4.CM0387.340.r2.d	UGT73F10	chr4.CM0739.7_at	0
chr4.CM0387.390.r2.d	UGT73F11	chr4.CM0739.20_at	0
chr4.CM0387.370.r2.d	UGT73F12	chr4.CM0739.17_at	0
chr4.CM0387.270.r2.d	UGT73F8	chr4.CM0739.4_at	0
chr4.CM0387.360.r2.d	UGT73F9	chr4.CM0739.16_at	0
LjT01K14.20.r2.d	UGT74K1	TM1443.6_at	0
LjSGA_015360.1	UGT74K3	Ljwgs_015360.1_at	0
chr1.CM0393.980.r2.m	UGT74X3	Ljwgs_051780.1_at	0
chr1.CM0393.1120.r2.d	UGT74X4	chr4.CM0165.11_at	0
chr4.CM0044.490.r2.m	UGT75L15	chr4.CM0044.55_at	0
chr6.LjT14E02.150.r2.d	UGT76J1	TM1445.31_at	0
chr6.CM0055.510.r2.m	UGT78K9	chr6.CM0055.30_at	0
chr3.LjT14J20.170.r2.m	UGT79B12	chr3.TM0388.25_at	0
chr3.LjT14J20.160.r2.m	UGT79B13	chr3.TM0388.23_at	0
chr6.CM0114.250.r2.m	UGT79B14	TM1489.2_at	0
chr5.CM0052.810.r2.m	UGT80B12	chr5.CM0052.35_at	0
chr4.CM0219.90.r2.d	UGT81A5	chr4.CM0219.14_at	0
chr1.CM0410.140.r2.m	UGT83G2	chr1.CM0410.56_at	0
chr1.CM0410.100.r2.m	UGT83G5	chr1.CM0410.64_at	0
chr1.CM0410.110.r2.m	UGT83G6	chr1.CM0410.62_at	0
chr1.CM0410.130.r2.m	UGT83G7	chr1.CM0410.62_at	0
chr1.CM0410.150.r2.m	UGT83G8	chr1.CM0410.54_at	0

chr2.CM1882.280.r2.a	UGT83J1	Ljwgs_022207.1_at	0
chr2.CM1882.260.r2.a	UGT83K1	Ljwgs_060242.1_at	0
chr4.CM0528.110.r2.d	UGT84H2	CM0528.29_at	0
chr5.CM0200.3230.r2.m	UGT84L1	chr5.CM0200.54_at	0
LjSGA_003166.2	UGT85A11	Ljwgs_003166.2_at	0
chr2.CM0124.440.r2.m	UGT85H1	Ljwgs_091755.2_at	E-172
chr3.LjT45I18.30.r2.d	UGT85K2	TM1472.14_at	0
chr3.CM0241.610.r2.m	UGT85K3	Ljwgs_024479.1_s_at	0
chr2.LjT09A09.80.r2.d	UGT87E1	chr2.TM0889.12_at	0
chr1.CM1868.190.r2.m	UGT89B6	Ljwgs_032790.2_at	0
chr2.CM0124.1460.r2.m	UGT89D1	chr2.CM0124.5_at	0
chr6.CM0314.440.r2.m	UGT91H1	chr6.CM0314.44_at	0
chr6.CM0314.450.r2.m	UGT91H2	chr6.CM0314.45_at	0
chr6.CM0314.490.r2.m	UGT91H3	chr6.CM0314.48_at	0
LjSGA_028050.1	UGT91N2	Ljwgs_028050.1_at	0
chr3.LjB09K03.30.r2.m	UGT92A2	chr3.BM1720.10_at	0
LjT09A12.80.r2.m	UGT93A10	TM0748.5_x_at	0
chr5.CM0278.660.r2.m	UGT93A6	TM0748.2_x_at	E-108
LjT09A12.10.r2.m	UGT93A7	TM0748.2_x_at	0
LjT09A12.20.r2.m	UGT93A8	TM0748.2_x_at	0
LjT09A12.60.r2.m	UGT93A9	TM0748.9_x_at	0

Note: e-value between nucleotide sequences of genes and the probe sets

Table S3. Sequence identity among 12 members of UGT72 family in *L. japonicus*

	Genbank Accession No.	UGT72AD1	UGT72AD2	UGT72AE1	UGT72AF1	UGT72AG1	UGT72AH1	UGT72B4	UGT72K1	UGT72L6	UGT72V2	UGT72V3	UGT72Z2
**UGT72AD1**	**AP009657**	100	79	33	32	36	48	43	33	50	33	34	50
**UGT72AD2**	**KT895082**		100	35	34	33	46	43	33	49	35	35	46
**UGT72AE1**	**KP410265**			100	50	43	36	42	37	38	41	43	37
**UGT72AF1**	**KT895083**				100	41	33	41	35	37	39	39	36
**UGT72AG1**	**KT895084**					100	36	43	39	39	44	44	37
**UGT72AH1**	**KT895085**						100	46	35	53	37	38	55
**UGT72B4**	**AK336772**							100	36	52	39	39	49
**UGT72K1**	**KP410266**								100	35	41	41	36
**UGT72L6**	**KT895087**									100	39	38	57
**UGT72V2**	**AP010407**										100	84	39
**UGT72V3**	**KT895088**											100	40
**UGT72Z2**	**KP410264**												100

Table S4. Sequence identity of 12 members of UGT72 family with their top orthologs

Gene ID	Genbank Accession No.	Plant Species	Genbank Accession No.	Identity
**UGT72AD1**	**AP009657**	*Glycine soja*	KHN34494.1	64%
**UGT72AD2**	**KT895082**	*Glycine soja*	KHN34494.1	63%
**UGT72AE1**	**KP410265**	*Glycine max*	XP_003524274.1	61%
**UGT72AF1**	**KT895083**	*Vitis vinifera*	XP_002281768.1	52%
**UGT72AG1**	**KT895084**	*Pueraria montana* var. *lobata*	ADV71360.1	72%
**UGT72AH1**	**KT895085**	*Medicago truncatula*	XP_003626718.1	68%
**UGT72B4**	**AK336772**	*Cicer arietinum*	XP_004494807.1	78%
**UGT72K1**	**KP410266**	*Glycine max*	XP_003521983.1	65%
**UGT72L6**	**KT895087**	*Phaseolus vulgaris*	XP_007134372.1	73%
**UGT72V2**	**AP010407**	*Glycine max*	XP_003527240.1	67%
**UGT72V3**	**KT895088**	*Glycine max*	XP_003527240.1	64%
**UGT72Z2**	**KP410264**	*Glycine max*	XP_003532192.1	73%

Table S5. Docking statistic and percentage of substrate converted for the 12 recombinant UGT proteins in *L. japonicus*

Names	Receptor-ligand (UDPG-Kaempferol)		Percentage of substrate converted (%)												
	Score	Area	Ka	Qu	My	Ap	Lu	Dai	Gen	Cy	Del	Pel	Ca	Epi	Egc
UGT72AD1	4088	452.70	6	80	17	-	-	-	-	-	-	-	-	-	-
UGT72AF1	3886	503.60	-	-	-	56	-	13	34	-	-	-	-	-	-
UGT72AH1	4260	494.50	19	42	63	-	-	-	-	-	-	-	-	-	-
UGT72V3	3966	467.30	12	38	15	-	6	11	17	-	-	-	-	-	-
UGT72Z2	4022	517.30	42	7	24	-	-	-	-	-	-	-	-	-	-
UGT72AD2	3976	445.30	-	-	-	-	-	-	-	-	-	-	-	-	-
UGT72AE1	3970	463.00	-	-	-	-	-	-	-	-	-	-	-	-	-
UGT72AG1	3718	498.40	-	-	-	-	-	-	-	-	-	-	-	-	-
UGT72B4	3720	493.20	-	-	-	-	-	-	-	-	-	-	-	-	-
UGT72K1	3958	484.40	-	-	-	-	-	-	-	-	-	-	-	-	-
UGT72L6	4236	484.60	-	-	-	-	-	-	-	-	-	-	-	-	-
UGT72V2	3794	477.70	-	-	-	-	-	-	-	-	-	-	-	-	-

Note: Ka, kaempferol; Qu, quercetin; My, myricetin; Ap, apigenin; Lu, luteolin; Dai, daidzein; Gen, genistein; Cy, cyanidin; Del, delphinidin; Pel, pelargonidin; Ca, catechin; Epi, epicatechin; Egc, epigallocatechin.

Score: Geometric shape complementarity score.

Area: Approximate interface area of the complex.

Percentage of substrate converted represents the means of triplicate enzymatic assays.

-: Not detected.

Table S6. Primer sequence used in the present study

Gene	Primer name	Primer sequence (5' to 3')	Gene Length (bp)	Protein Length (aa)
UGT72AD1	UGT72AD1BF	cgggatccATGGAGACCACAACAACAACTC	1449	482
	UGT72AD1SR	gtgtcgacTTAGTTCCCTTCCCCTTCAACC		
	UGT72AD1qRTR	TTGCAGGGTCAGAGGTTTGA	256	/
UGT72AD2	UGT72AD2XF	ttttctagaATGGGGTTTTACGATGTGGGT	1551	516
	UGT72AD2HR	tttaagcttTTAGACCGCTGACCTTTTCCA		
UGT72AE1	UGT72AE1BF	cgggatccATGACGGAATCCACGAAACCG	1437	478
	UGT72AE1SR	gtgtcgacTTATGGGGCTTCATTGCACTGGC		
UGT72AF1	UGT72AF1 EF	aaagaatccATGGTGTTCGATGCCAAGAAAC	1410	469
	UGT72AF1BR	aaaggatccCTAATTAATCATACGGACATGA		
UGT72AG1	UGT72AG1BF	aaaggatccATGTCTCTTGGACATGGTCGA	1527	508
	UGT72AG1XR	aaatctagaTCACACTTTTGCCGTTGAAAAC		
UGT72AH1	UGT72AH1XF	ttttatagaATGGAAGCAAGAACTCACATAG	1425	474
	UGT72AH1HR	aaaaagcttTTAGTCTTGTGTGATTGTGCC		
	UGT72AH1qRTR	GTGACTTGGAAATTGTCTTG	116	/
UGT72B4	UGT72B4CF	cgggatccATGGAAGAACAATTGAAACTAGTAG	1446	481
	UGT72B4SR	gtgtcgacTTACATGGTTACCGTAACTTTGC		
UGT72K1	UGT72K1EF	tttgaattcATGGCTGCCTCAAACTCTCA	1464	487
	UGT72K1BR	aaaggatccTCATAACGCACCATTTGCCT		
UGT72L6	UGT72L6BF	cgggatccATGGAGAAGAAAATTCACATTGC	1422	473
	UGT72L6SR	gtgtcgacTACCAATTTTTTCCACTTGAGTA		

UGT72V2	UGT72V2BF	cgggatccATGGAGGACTTGCACAAGCAC	1413	470
	UGT72V2SR	gtgtcgacTCATCCTTCGATCATTTTCGCTA		
UGT72V3	UGT72V3XF	ttttctagaATGGAAGACATGCAGAACCAA	1425	474
	UGT72V3HR	aaaaagcttTCATCCTCCGATAATAATTTTCG		
UGT72Z2	UGT72Z2BF	cgggatccATGGCCAAAACAATTCACATAGC	1470	489
	UGT72Z2SR	gtgtcgacCTTTAGTGCCAGGAAGTTTTCAA		
	UGT72Z2qRTR	CTGCACTGCAGGGGAAACA	252	/
LJEF1α	LJEF1αF	TGTGAAGGATCTCAAGCGTG	103	/
	LJEF1αR	GTATGGCAATCAAGGACTGG		
LjUBQ10	LjUBQ10qRTF	TTCACCTTGTGCTCCGTCTTC	66	/
	LjUBQ10qRTR	AACAACAGCACACACAGACAATCC		
Ljβ-actin	Ljβ-ACTINF	GCATTGTTGGTCGTCCTCGT	79	/
	Ljβ-ACTINR	TGTGCCTCATCCCCAACATA		
AtP2Pa	P2PaF	TATCGGATGACGATTCTTCGTGCAG	173	/
	P2PaR	GCTTGGTCGACTATCGGAATGAGAG		

Note: Lowercase letters indicate linker nucleotides containing restriction sites for cloning.

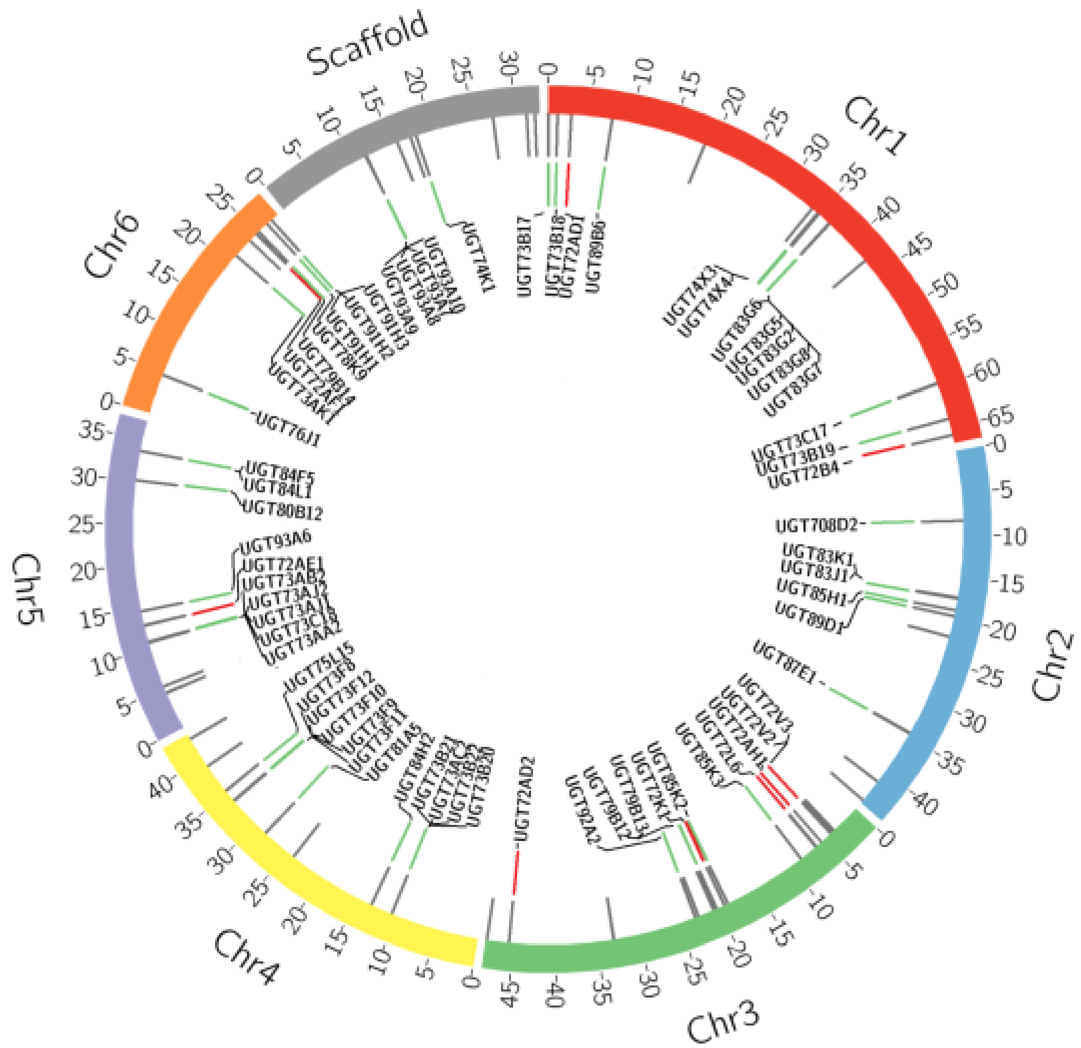

Figure S1. Genomic distribution of 71 full-length *UTG* genes identified in the *L. japonicus* genome.

Chromosomal distribution of *UTGs* identified in *L. japonicus*. Six chromosomes and scaffold were indicated in different color. All predicated UGTs were indicated by gray sticks in the outer lines and full-length UGTs were indicated by green sticks in the inner lines. The UGTs from UGT72 family were indicated in red.

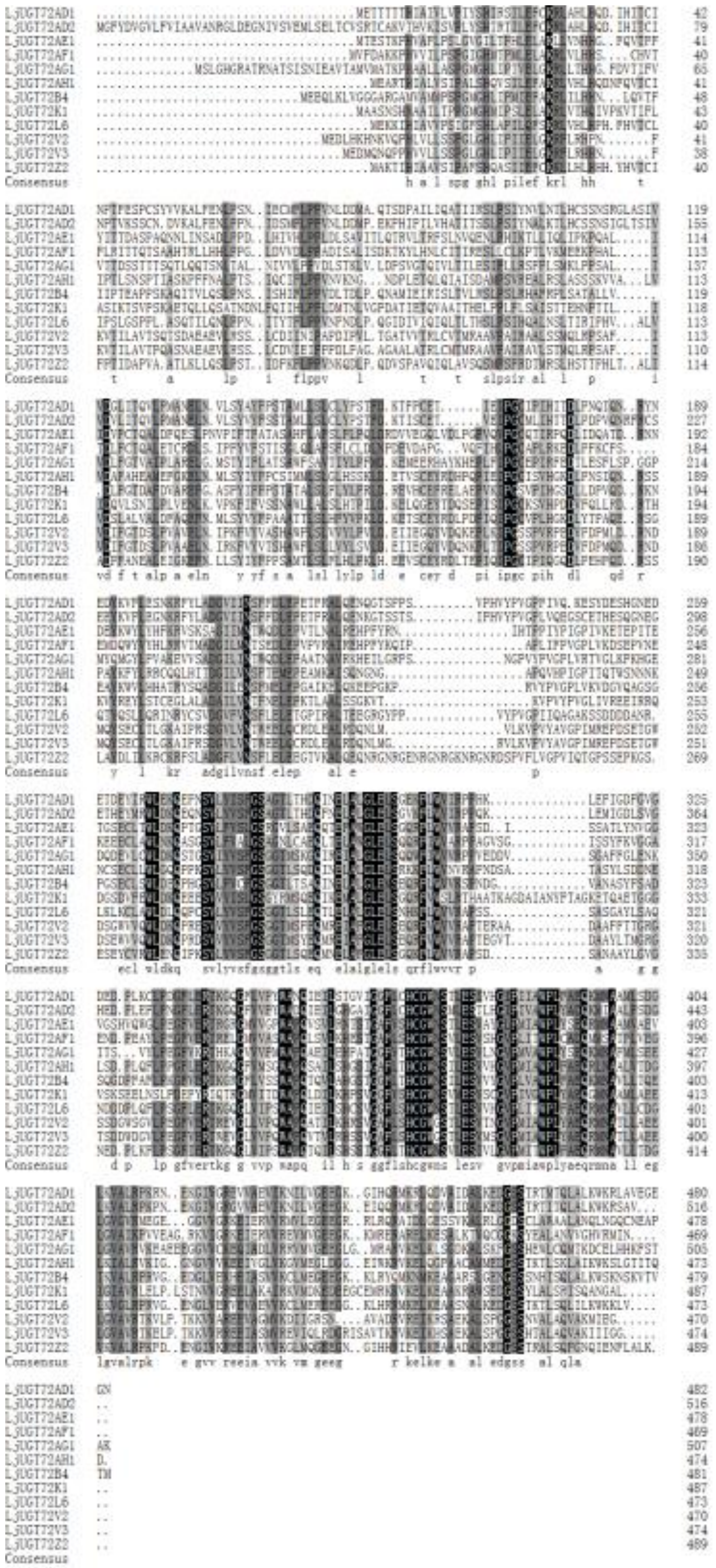

Figure S2. Multiple sequence alignment of twelve members of UGT72 family in *L. japonicus*.

Identical residues are highlighted in black background and similar residues are highlighted in gray background.

A

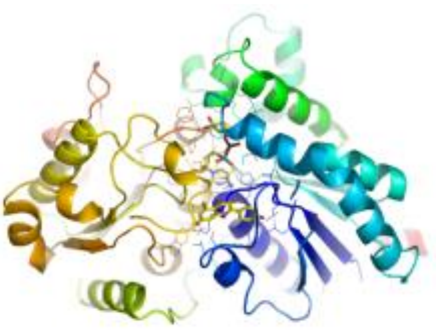

UGT72AD1

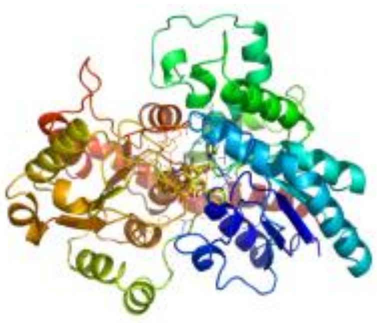

UGT72AD2

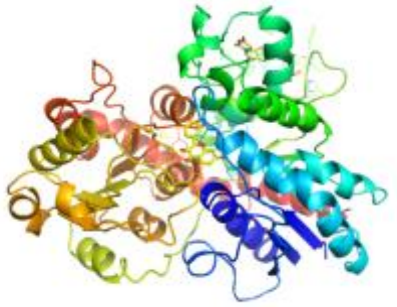

UGT72AE1

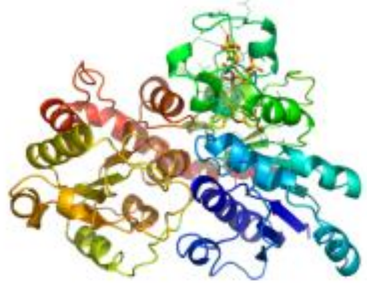

UGT72AF1

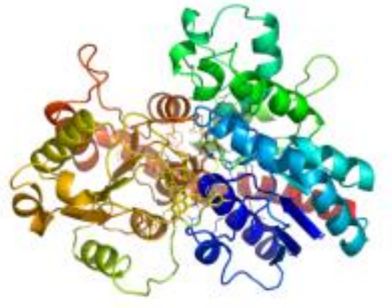

UGT72AG1

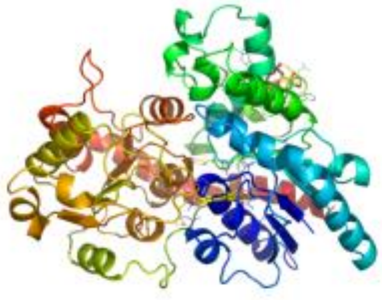

UGT72AH1

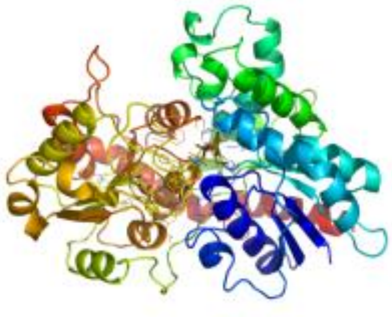

UGT72B4

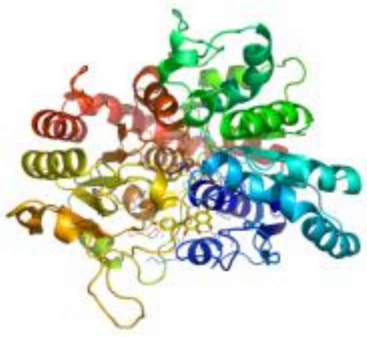

UGT72K1

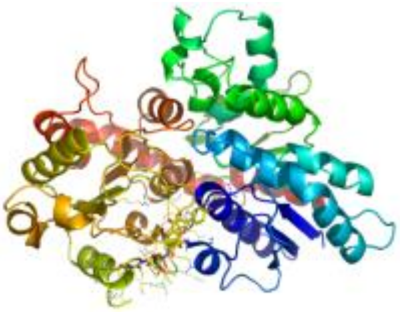

UGT72L6

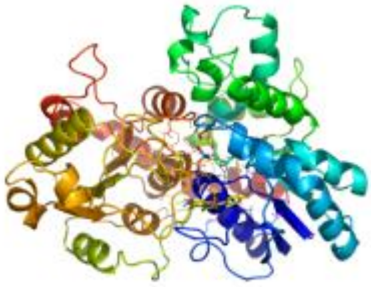

UGT72V2

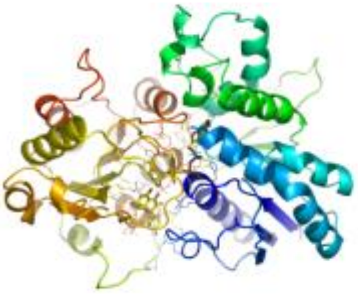

UGT72V3

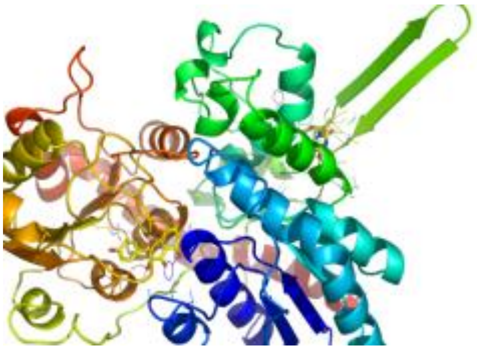

UGT72Z2

B

UGT72AF1

UGT72V3

UGT72L6

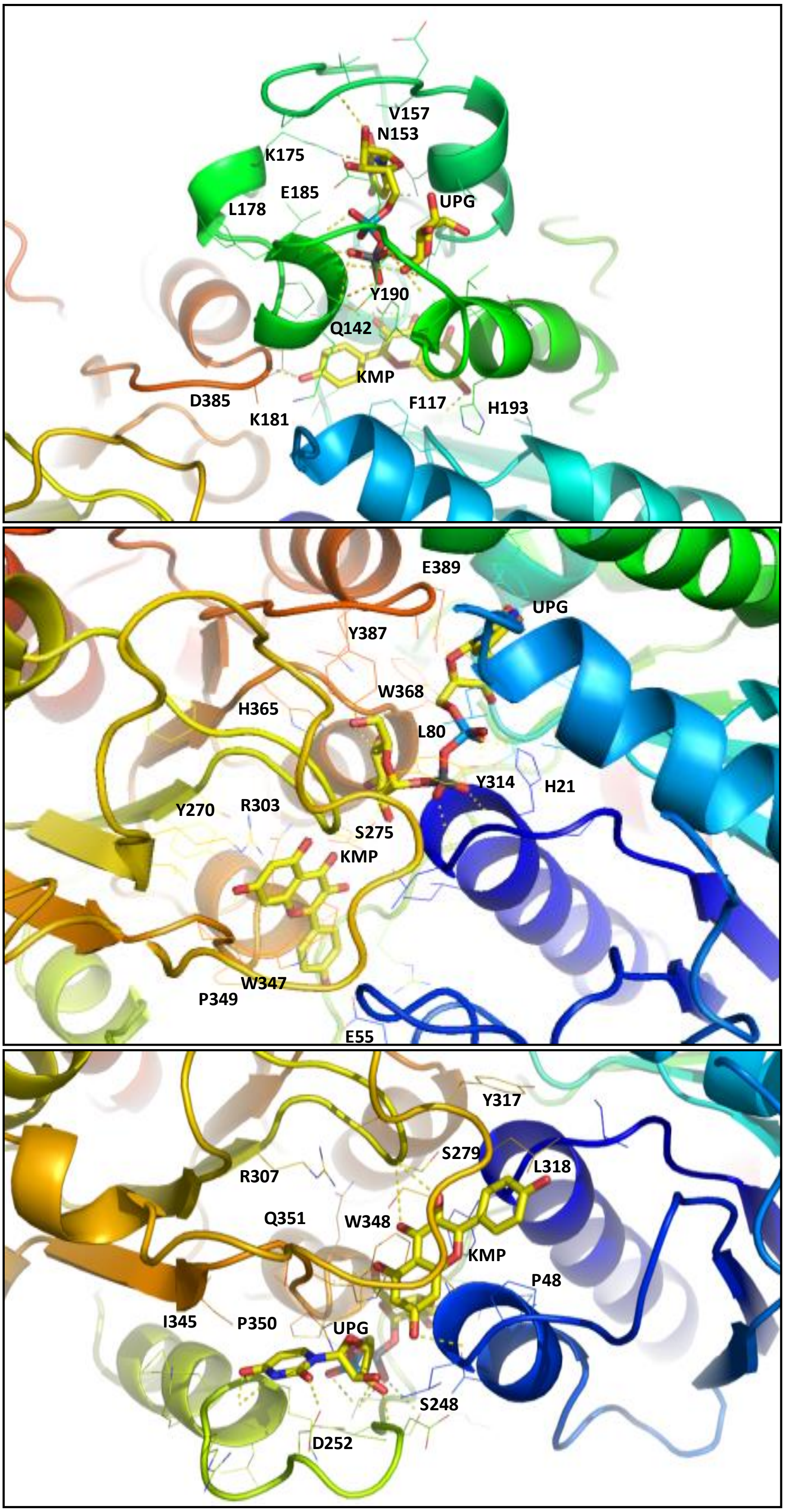

C

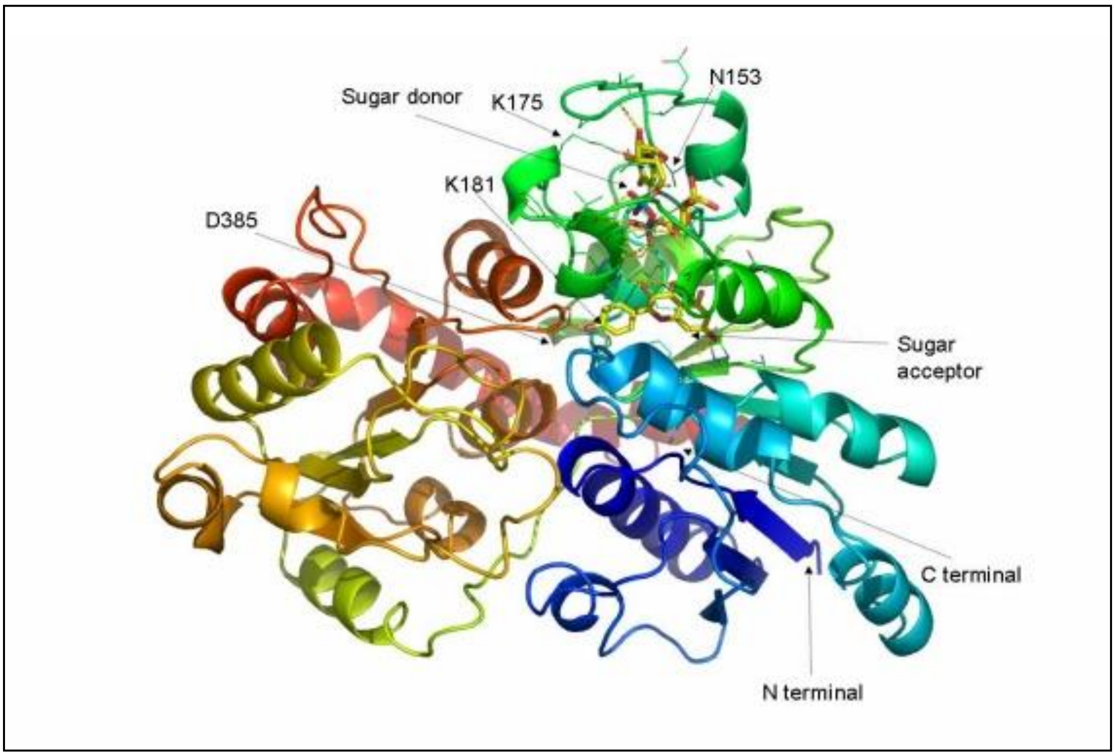

UGT72AD1

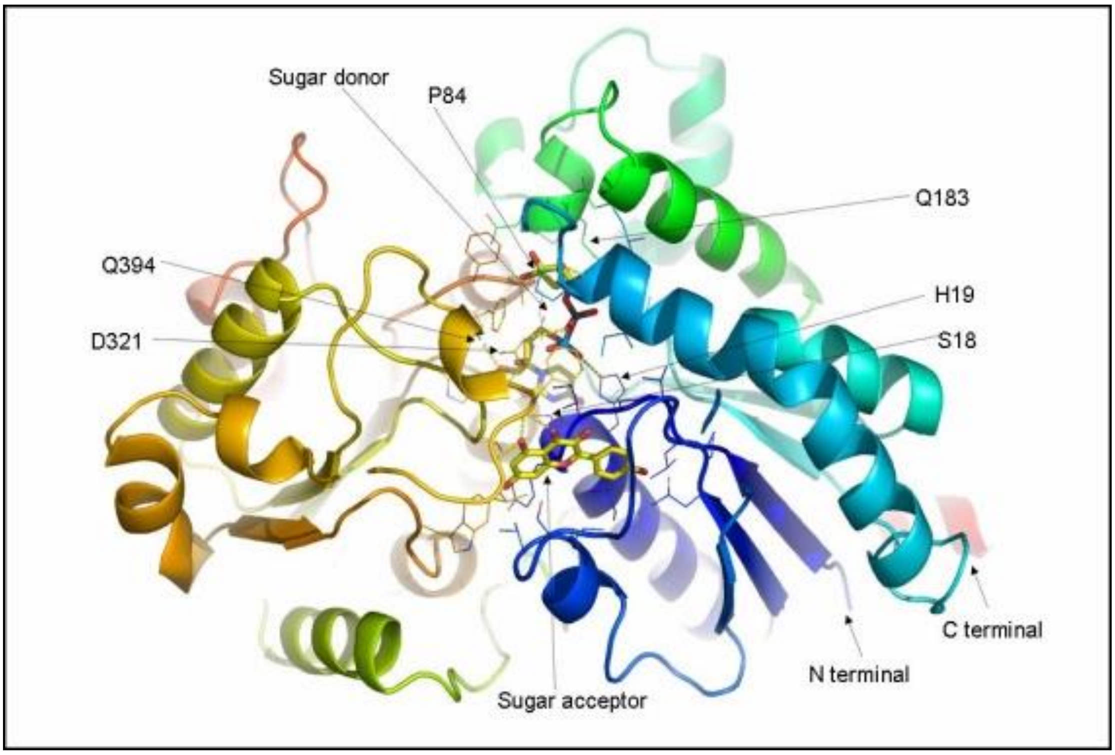

UGT72AF1

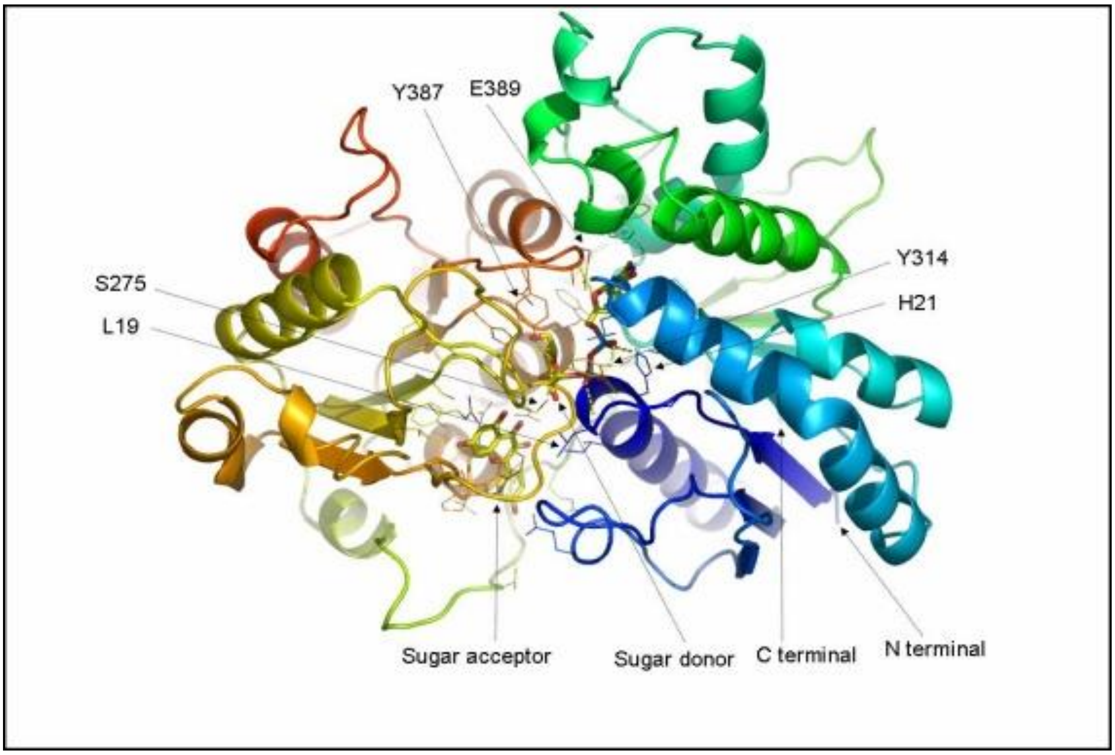

UGT72V3

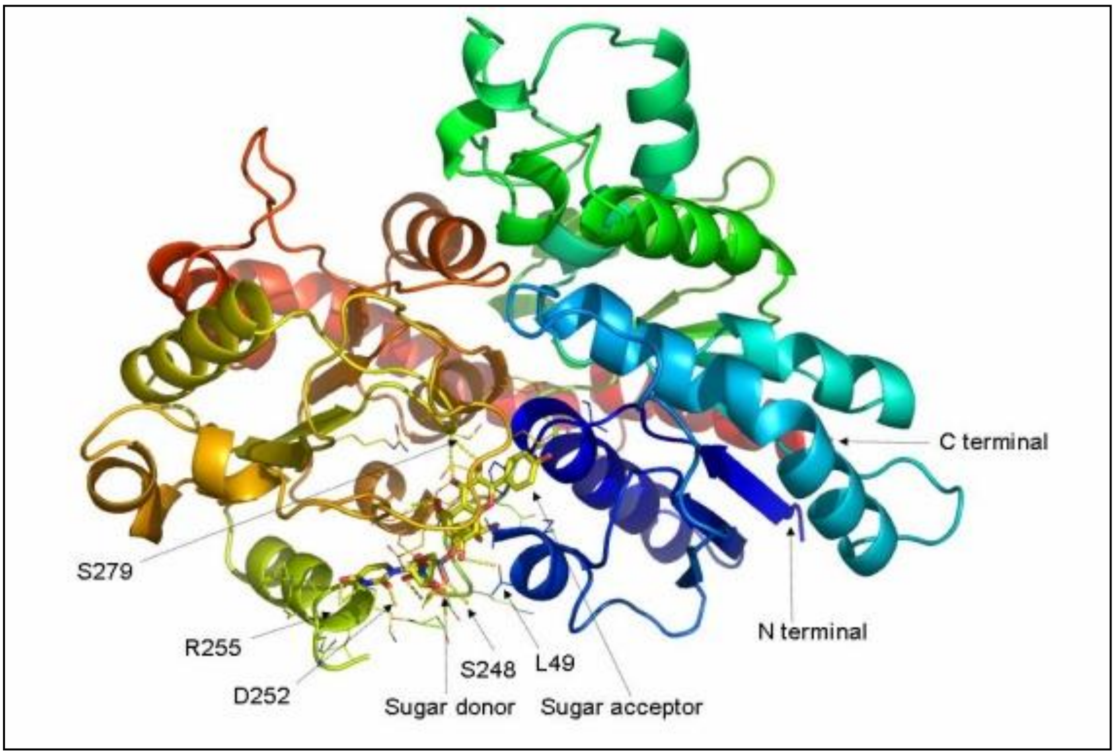

UGT72L6

Figure S3. Homology docking of twelve members of UGT72 family in *L. japonicus*.

(A) The structural models docked with UDP-glucose (UPG) and kaempferol for twelve UGTs of UGT72 family.

(B) The structural models of three representative UGTs docked with UDP-glucose and a flavonol (kaemperol). From top to bottom: UGT72AF1, UGT72V3 and UGT72L6.

(C) Highlighted structural models of UGT72AD1, UGT72AF1, UGT72V3 and UGT72AL6 docked with UDP-glucose (UPG) and kaempferol. The amino acids in the form of ligands were labeled.

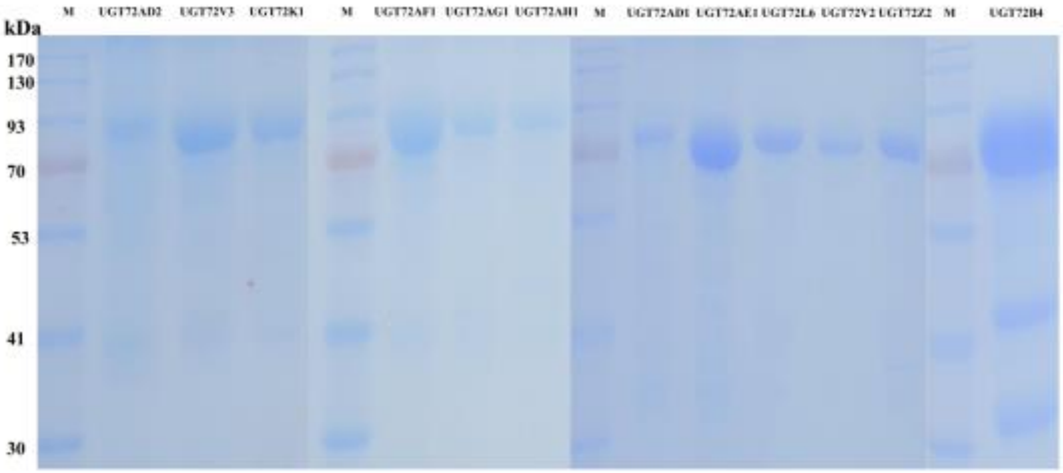

Figure S4. Twelve purified recombinant UGT proteins on denaturing PAGE.

M, prestained protein marker with sizes of 170, 130, 93, 70, 53, 41, 30 kDa.

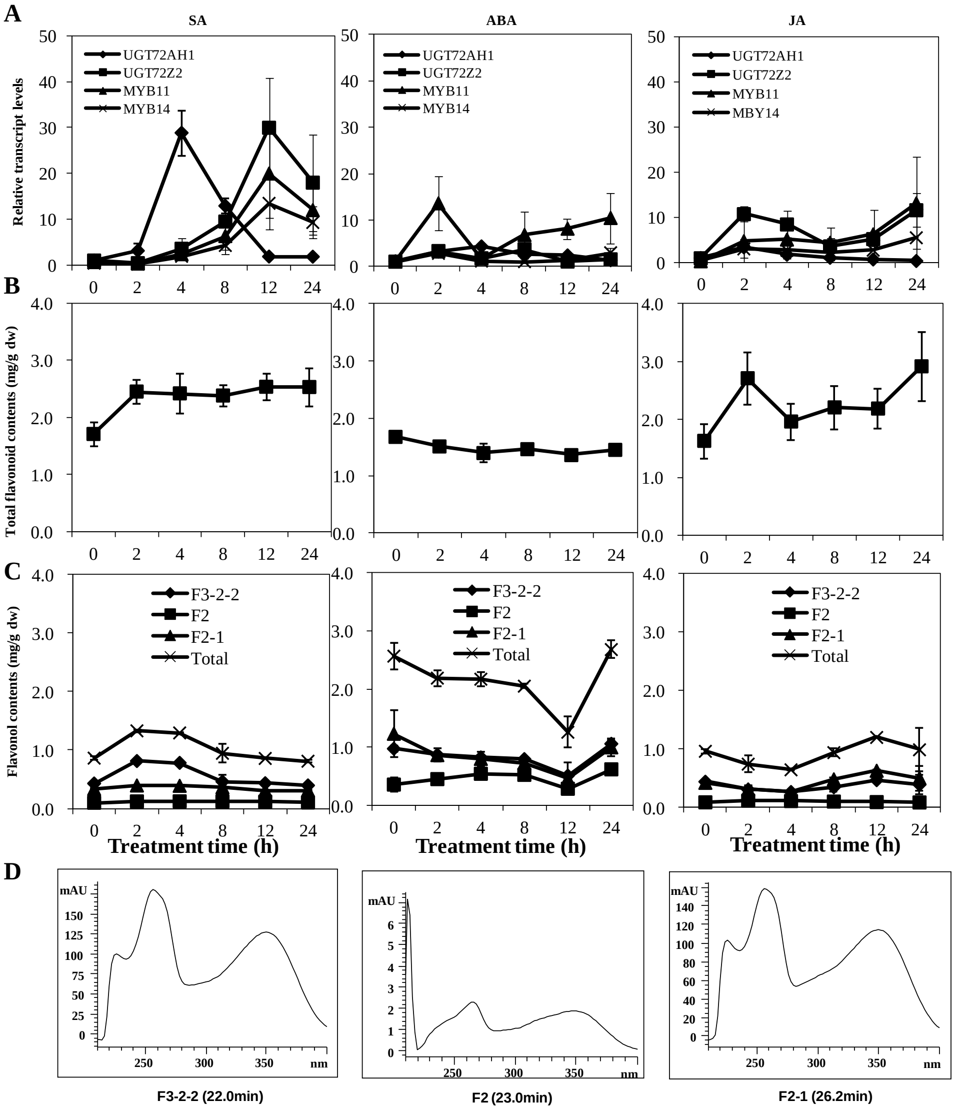

Figure S5. Effects of SA, ABA and JA treatments on *L. japonicus* hairy roots.

(A) Relative transcript levels of *UGT72AH1* and *UGT72Z2*, together with two transcription factors *MYB11* and *MYB14* at different time points. Left, SA treatment; Middle, ABA treatment; Right, JA treatment.

(B) The content of total flavonoids in *L. japonicus* hairy roots by SA, ABA and JA treatments.

(C) The content of individual flavonol in *L. japonicus* hairy roots by SA, ABA and JA treatments.

Values represent the means and standard deviations of triplicate treatments.

(D) UV spectrum of flavonol glucosides F3-2-2, F2 and F2-1.

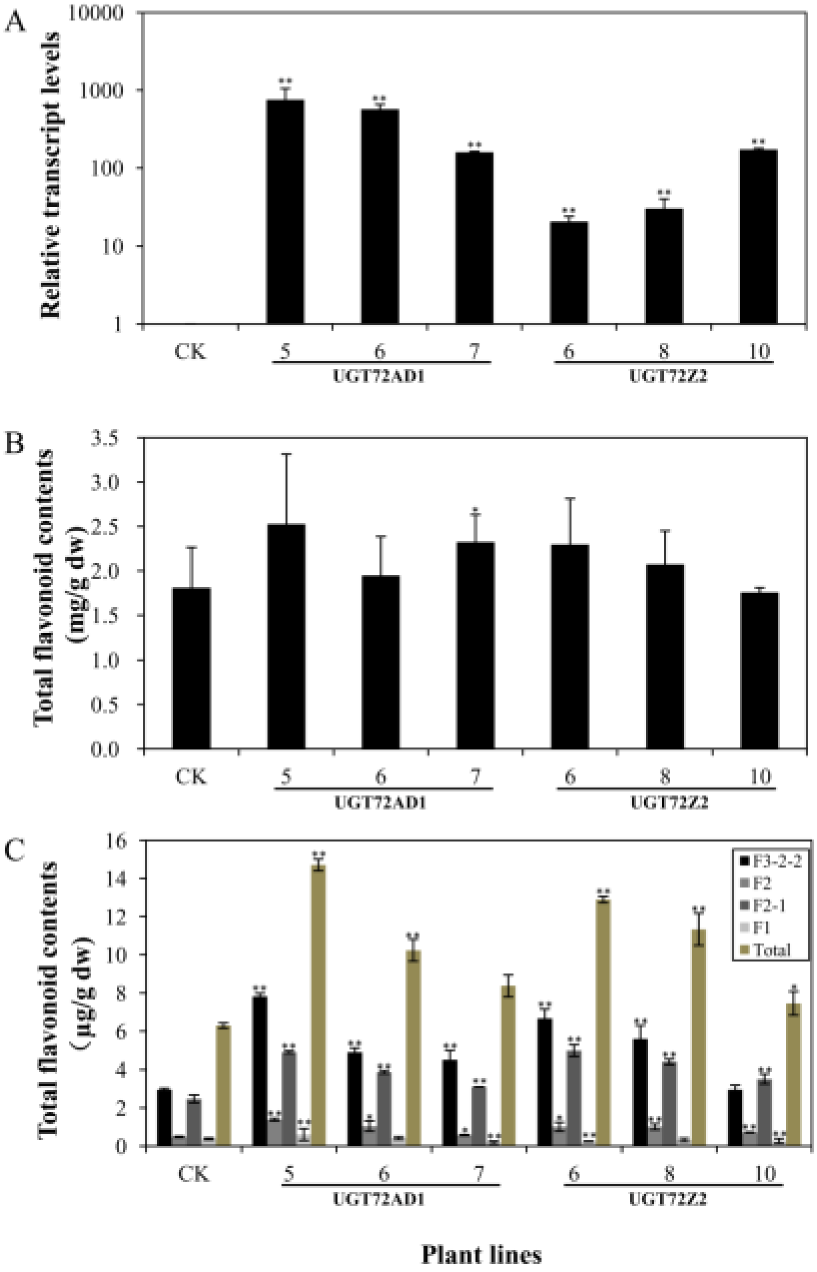

Figure S6. Over-expression of *UGT72AD1* and *UGT72Z2* genes in hairy roots of *L. japonicus*.

(A) Relative transcript levels of *UGT72AD1* and *UGT72Z2* genes in hairy roots detected by qRT-PCR analysis.

(B) Total flavonoid contents in *L. japonicus* hairy root lines over-expressing *UGT72AD1* and *UGT72Z2* genes.

(C) Flavonol contents in *L. japonicus* hairy root lines over-expressing *UGT72AD1* and *UGT72Z2* genes.

Values represent the means and standard deviations of triplicate analytical replicates from independent transgenic lines and control. The data were statistically evaluated using Student's *t* test (**$P<0.01$,* $P<0.05$).

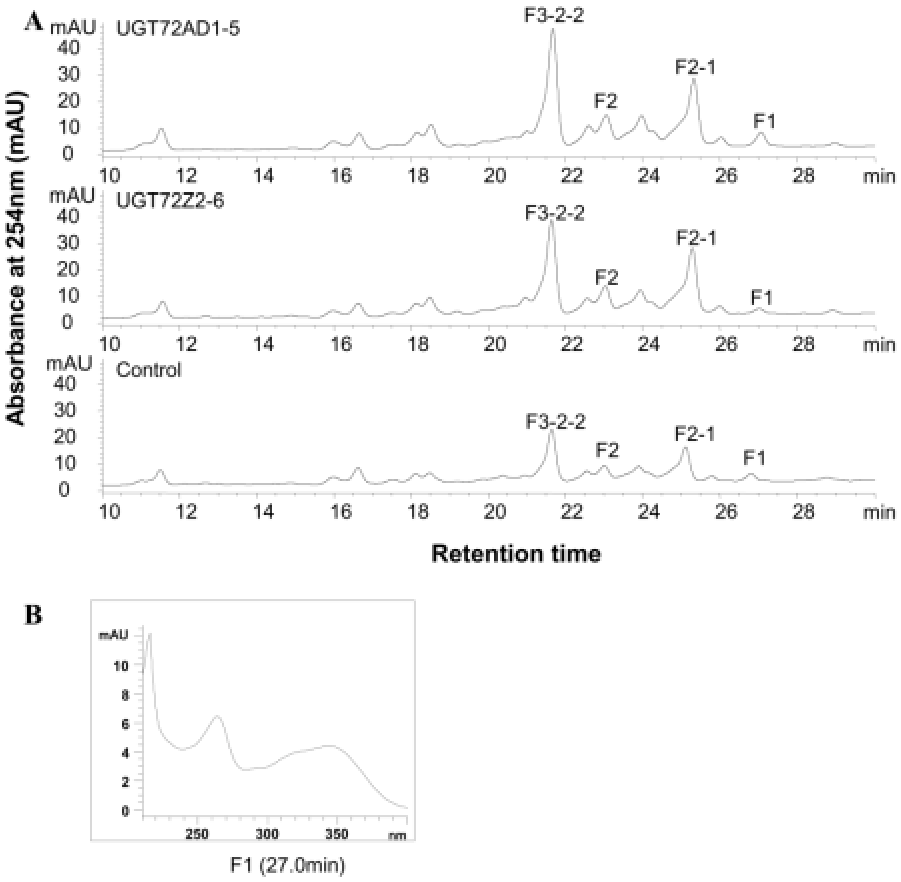

Figure S7. Representative HPLC chromatograms of *UGT72AD1* and *UGT72Z2* over- expressing lines in hairy roots of *L. japonicus*.

(A) HPLC chromatograms were obtained from methanolic extracts of transgenic hairy root lines.

(B) UV spectrum of flavonol glucoside F1.

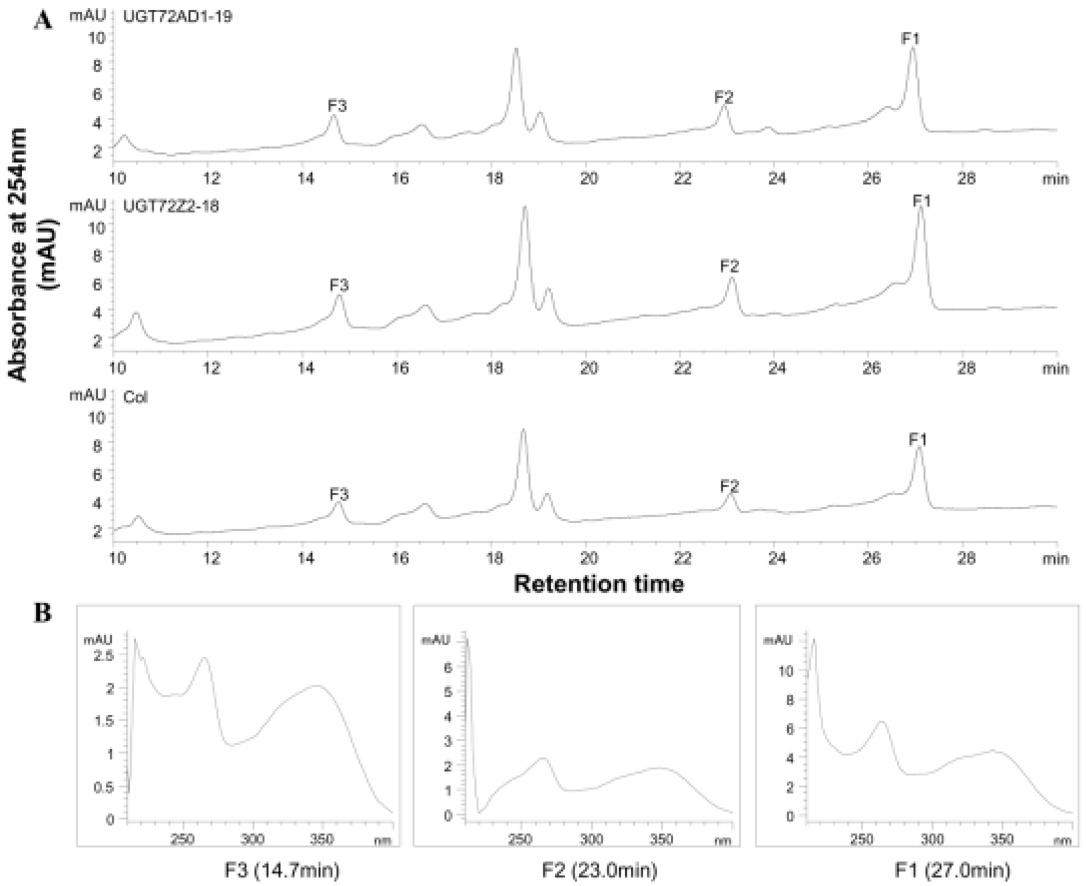

Figure S8. Representative HPLC chromatograms of *UGT72AD1* and *UGT72Z2* over-expressing lines in seedlings of *A. thaliana*.

(A) The HPLC chromatograms were obtained from methanolic extracts of transgenic *A. thaliana* seedlings of lines UGT72AD1-19 and UGT72Z2-18 together with wild type Col as control.

(B) UV spectrum of flavonoid compounds F3, F2 and F1.

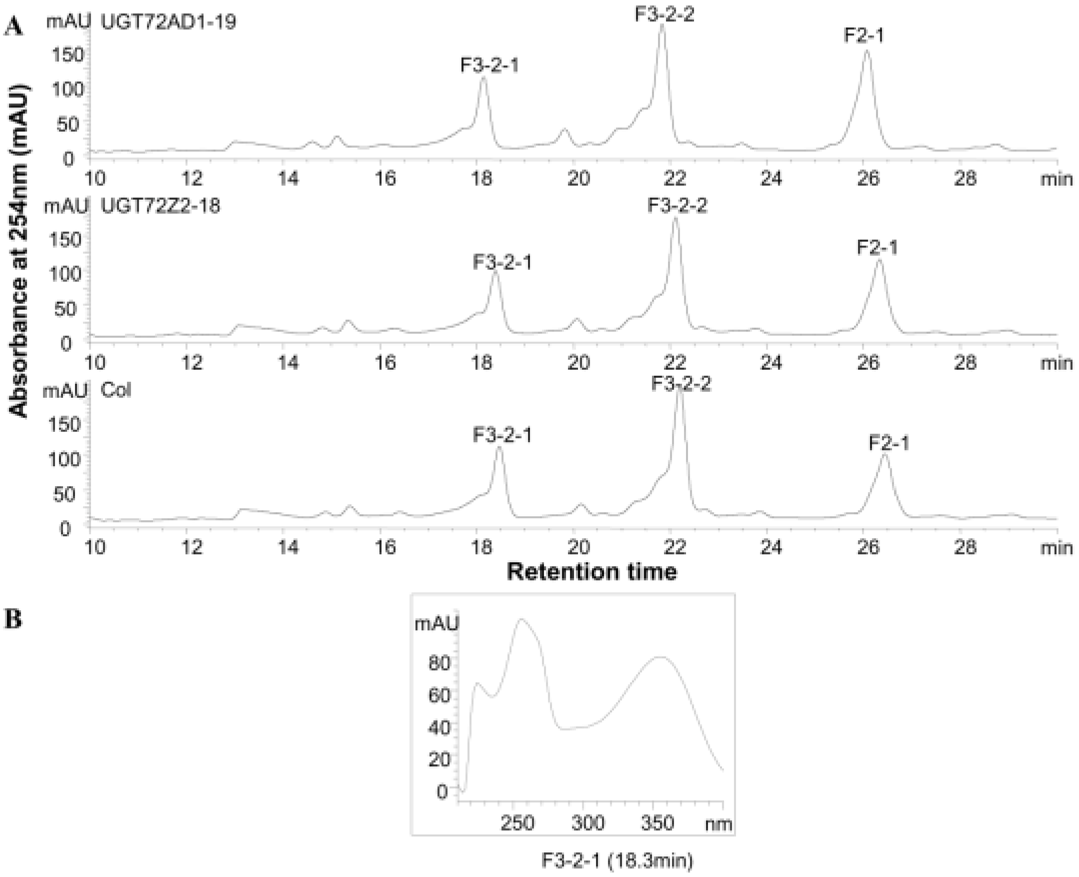

Figure S9. Representative HPLC chromatograms of *UGT72AD1* and *UGT72Z2* over-expression lines in seeds of *A. thaliana*.

(A) The HPLC chromatograms were obtained from methanolic extracts of transgenic *A. thaliana* seeds of lines UGT72AD1-19 and UGT72Z2-18 together with wild type Col as control.

(B) UV spectrum of flavonoid compounds F3-2-1.

Accession numbers:

Gene names, plant species and GenBank accession numbers are as follows: AmF7GAT, BAG31945.1 (*Antirrhinum majus*); AmF7GlcT, BAG31950.1 (*Antirrhinum majus*); At3AraT, NP_197205.1 (*A. thaliana*); AtF3G7GT, NP_181217.1 (*A. thaliana*); AtF3GlcT, NP_197297.1 (*A. thaliana*); AtF3RhaT, NP_564357.1 (*A. thaliana*); AtF5GlcT, NP_193146.1 (*A. thaliana*); AtF7GlcT, NP_567955.1 (*A. thaliana*);

AtF7RhaT, NP_563756.1 (*A. thaliana*); Cs3GlcT, AAS00612 (*Citrus sinensis*); Dc3GT1, BAD52003.1 (*Dianthus caryophyllus*); FaGT6, Q2V6K0.1 (*Fragaria vesc*a subsp. vesca); GeIF7GlcT, BAC78438.1 (*Glycyrrhiza echinata*); Gh5GT, BAA36423 (*Glandularia × hybrida*); GhA5GlcT, Q9ZR25.1 (*Glandularia × hybrida*); GmIF7GlcT, NP_001235161.1 (*G. max*); GT04F14, ADV71364.1 (*P. montana* var. lobata); GT12D15, ADV71367.1 (*P. montana* var. lobata); Hg3GT, P14726 (*Hordeum vulgare*); Ih3GT, BAD83701.1 (*Iris × hollandica*); Ih5GT, BAD06874 (*Iris × hollandica*); Lb7GlcT, BAG80536.1 (*Lycium barbarum*); Mt7GT, AAW56091 (*M. truncatula*); Per5GT, BAA36421 (*Perilla frutescens* var. crispa); PfA5GlcT, Q9ZR27.1 (*Perilla frutescens*); PhA5GlcT, BAA89009.1 (*Petunia × hybrida*); RhA53GlcT, Q4R1I9.1 (*Rosa hybrid* cultivar); SbF7GlcT, BAA83484.1 (*Scutellaria baicalensis*); SlF7GAT, BAG31946.1 (*Scutellaria laeteviolacea* var. yakusimensis); ThA5GlcT, BAC54093 (*Torenia hybrid*); UGT72AD1, AP009657 (*L. japonicus*); UGT72AD2, KT895082 (*L. japonicus*); UGT72AE1, KP410265 (*L. japonicus*); UGT72AF1, KT895083 (*L. japonicus*); UGT72AG1, KT895084 (*L. japonicus*); UGT72AH1, KT895085 (*L. japonicus*); UGT72B3, AEE27284.1 (*A. thaliana*) UGT72B4, AK336772 (*L. japonicus*); UGT72B18, AGU14071.1 (*C. arietinum*); UGT72K1, KT895087 (*L. japonicus*); UGT72L1, CC38470.1 (*M. truncatula*); UGT72L6, KP410266 (*L. japonicus*); UGT72V2, AP010407 (*L. japonicus*); UGT72V3, KT895088 (*L. japonicus*); UGT72Z2, KP410264 (*L. japonicus*); UGT78K1, ADC96620 (*G. max*); VmF3GalT, BAA36972.1 (*Vigna mungo*); VvF3GlcT, P51094.2 (*V. vinifera*); ZmF3GT, P16165.1 (*Z. mays*).
